# Supplementary material for: “Trying to remain calm…but I do reach my limit sometimes”: An exploration of the meaning of gentle parenting
Source: PLoS One. 2024 Jul 31;19(7):e0307492. doi: 10.1371/journal.pone.0307492 (PMC11290678; doi:10.1371/journal.pone.0307492)
Supplement: Attachment — Submitted filename: PONE-D-24-07088_R1 editor.pdf [file pone.0307492.s004.pdf]

PLOS ONE

“Trying to Remain Calm…But I do Reach my Limit Sometimes”: An Exploration of the Meaning of Gentle Parenting

--Manuscript Draft--

**Manuscript Number:**	PONE-D-24-07088R1
**Article Type:**	Research Article
**Full Title:**	“Trying to Remain Calm…But I do Reach my Limit Sometimes”: An Exploration of the Meaning of Gentle Parenting
**Short Title:**	An Exploration of the Meaning of Gentle Parenting
**Corresponding Author:**	Anne Elizabeth Pezalla, Ph.D.<br>Macalester College<br>St. Paul, UNITED STATES
**Keywords:**	parenting; co-parenting; Parenting stress; parenting burnout; authoritative parenting; indulgent parenting; early childhood development.
**Abstract:**	Raising young children has always been hard, but evidence suggests that it may be getting harder. The isolation of the pandemic, the pressures to fulfill exacting parenting standards, and the explosion of “expert” parenting advice on social media have fueled the rise of “gentle parenting,” an approach that pivots away from older, discipline-heavy parenting typologies and which promises the development of happier, healthier children. Despite the popularity of gentle parenting, it has received no empirical scrutiny. The current study represents the first systematic investigation of what gentle parenting entails. Data were gathered from a sample (N = 100) of parents of at least one child between the ages of 2 and 7 from the Midwest, Southeast, and Southwest. Approximately half (n = 49) of the sample identified as “gentle parents.” Inductive analyses identified this approach as one that emphasizes high levels of parental affection and parents’ and children’s emotion regulation. Gentle parenting appears to be distinct from other established measures of parenting approaches in its emphasis on boundaries, yet the enactment of those boundaries is not uniform. Overall, gentle parents reported high levels of parenting satisfaction and efficacy, but a subset of gentle parents who were highly critical of themselves reported significantly lower levels of efficacy than the rest of the sample. Statements of parenting uncertainty and burnout were present in over one-third of the gentle parent sample. Implications are discussed for future research and increased support for those who identify as gentle parents.
**Order of Authors:**	Anne E. Pezalla, Ph.D.
	Alice J. Davidson, Ph.D.
**Opposed Reviewers:**	
**Response to Reviewers:**	Within this submission, we have supplied the following:<br><br>A rebuttal letter that responds to each point raised by our reviewers. Per the instructions we received with this feedback, we uplaoded this letter as a separate file labeled 'Response to Reviewers'.<br><br>A marked-up copy of our manuscript that highlights the changes we made to the original version. We uploaded this as a separate file labeled 'Pezalla - Davidson - PLOS - Track Changes'.<br><br>An unmarked version of our revised paper without tracked changes. We uploaded this as a separate file labeled 'Pezalla - Davidson - PLOS - Clean'.<br><br>Thank you!<br><br>~Annie
**Additional Information:**	
**Question**	**Response**

**Financial Disclosure**<br><br>Enter a financial disclosure statement that describes the sources of funding for the work included in this submission. Review the submission guidelines for detailed requirements. View published research articles from *PLOS ONE* for specific examples.<br><br>This statement is required for submission and **will appear in the published article** if the submission is accepted. Please make sure it is accurate.<br><br>**Funded studies**<br>Enter a statement with the following details:<br>• Initials of the authors who received each award<br>• Grant numbers awarded to each author<br>• The full name of each funder<br>• URL of each funder website<br>• Did the sponsors or funders play any role in the study design, data collection and analysis, decision to publish, or preparation of the manuscript?<br><br>Did you receive funding for this work?	Yes
Please add funding details.<br>as follow-up to "**Financial Disclosure**<br><br>Enter a financial disclosure statement that describes the sources of funding for the work included in this submission. Review the submission guidelines for detailed requirements. View published research articles from *PLOS ONE* for specific examples.<br><br>This statement is required for submission and **will appear in the published article** if the submission is accepted. Please make sure it is accurate.	Annie Pezalla received a $1250 faculty fund from Macalester College for this study. She used those funds to compensate participants.

**Funded studies**<br>Enter a statement with the following details:<br>• Initials of the authors who received each award<br>• Grant numbers awarded to each author<br>• The full name of each funder<br>• URL of each funder website<br>• Did the sponsors or funders play any role in the study design, data collection and analysis, decision to publish, or preparation of the manuscript?<br><br>Did you receive funding for this work?"	
Please select the country of your main research funder (please select carefully as in some cases this is used in fee calculation).<br>as follow-up to "**Financial Disclosure**<br><br>Enter a financial disclosure statement that describes the sources of funding for the work included in this submission. Review the submission guidelines for detailed requirements. View published research articles from *PLOS ONE* for specific examples.<br><br>This statement is required for submission and **will appear in the published article** if the submission is accepted. Please make sure it is accurate.<br><br>**Funded studies**<br>Enter a statement with the following details:<br>• Initials of the authors who received each award<br>• Grant numbers awarded to each author<br>• The full name of each funder<br>• URL of each funder website<br>• Did the sponsors or funders play any role in the study design, data collection and analysis, decision to publish, or preparation of the manuscript?<br><br>Did you receive funding for this work?"	UNITED STATES - US
**Competing Interests**	The authors have declared that no competing interests exist.

Use the instructions below to enter a competing interest statement for this submission. On behalf of all authors, disclose any competing interests that could be perceived to bias this work—acknowledging all financial support and any other relevant financial or non-financial competing interests.<br><br>This statement is required for submission and **will appear in the published article** if the submission is accepted. Please make sure it is accurate and that any funding sources listed in your Funding Information later in the submission form are also declared in your Financial Disclosure statement.<br><br>View published research articles from *PLOS ONE* for specific examples.<br><br>**NO authors have competing interests**<br><br>Enter: *The authors have declared that no competing interests exist.*<br><br>**Authors with competing interests**<br><br>Enter competing interest details beginning with this statement:<br><br>*I have read the journal's policy and the authors of this manuscript have the following competing interests: [insert competing interests here]*<br><br>* typeset	
**Ethics Statement**<br><br>Enter an ethics statement for this submission. This statement is required if the study involved:<br><br>• Human participants<br>• Human specimens or tissue<br>• Vertebrate animals or cephalopods<br>• Vertebrate embryos or tissues	IRB approval #23046 was granted from Macalester College on June 27, 2023.<br><br>Rollins College also reviewed our study and granted IRB approval, with Macalester College as the IRB of record with oversight over Rollins.<br><br>The IRB approval from both Macalester and Rollins have been submitted as supplemental documents.<br><br>Written consent was obtained from all participants (consent form has also been submitted as supplemental documents).

Powered by Editorial Manager® and ProduXion Manager® from Aries Systems Corporation

• Field research<br><br>Write "N/A" if the submission does not require an ethics statement.<br><br>General guidance is provided below. Consult the submission guidelines for detailed instructions. **Make sure that all information entered here is included in the Methods section of the manuscript.**	

**Format for specific study types**<br><br>**Human Subject Research (involving human participants and/or tissue)**<br>• Give the name of the institutional review board or ethics committee that approved the study<br>• Include the approval number and/or a statement indicating approval of this research<br>• Indicate the form of consent obtained (written/oral) or the reason that consent was not obtained (e.g. the data were analyzed anonymously)<br><br>**Animal Research (involving vertebrate animals, embryos or tissues)**<br>• Provide the name of the Institutional Animal Care and Use Committee (IACUC) or other relevant ethics board that reviewed the study protocol, and indicate whether they approved this research or granted a formal waiver of ethical approval<br>• Include an approval number if one was obtained<br>• If the study involved *non-human primates*, add *additional details* about animal welfare and steps taken to ameliorate suffering<br>• If anesthesia, euthanasia, or any kind of animal sacrifice is part of the study, include briefly which substances and/or methods were applied<br><br>**Field Research**<br><br>Include the following details if this study involves the collection of plant, animal, or other materials from a natural setting:<br>• Field permit number<br>• Name of the institution or relevant body that granted permission	
**Data Availability**<br><br>Authors are required to make all data underlying the findings described fully available, without restriction, and from the time of publication. PLOS allows rare exceptions to address legal and ethical concerns. See the PLOS Data Policy and FAQ for detailed information.	Yes - all data are fully available without restriction

Powered by Editorial Manager® and ProduXion Manager® from Aries Systems Corporation

A Data Availability Statement describing where the data can be found is required at submission. Your answers to this question constitute the Data Availability Statement and **will be published in the article**, if accepted.<br><br>**Important:** Stating 'data available on request from the author' is not sufficient. If your data are only available upon request, select 'No' for the first question and explain your exceptional situation in the text box.<br><br>Do the authors confirm that all data underlying the findings described in their manuscript are fully available without restriction?	
**Describe where the data may be found in full sentences. If you are copying our sample text, replace any instances of XXX with the appropriate details.**<br><br>• If the data are **held or will be held in a public repository**, include URLs, accession numbers or DOIs. If this information will only be available after acceptance, indicate this by ticking the box below. For example: *All XXX files are available from the XXX database (accession number(s) XXX, XXX.).*<br>• If the data are all contained **within the manuscript and/or Supporting Information files**, enter the following: *All relevant data are within the manuscript and its Supporting Information files.*<br>• If neither of these applies but you are able to provide **details of access elsewhere**, with or without limitations, please do so. For example:<br><br>*Data cannot be shared publicly because of [XXX]. Data are available from the XXX Institutional Data Access / Ethics Committee (contact via XXX) for researchers who meet the criteria for access to confidential data.*<br><br>*The data underlying the results presented in the study are available from (include the name of the third party*	All relevant data are within the manuscript and its supporting information files.

and contact information or URL).<br>• This text is appropriate if the data are owned by a third party and authors do not have permission to share the data.<br><br>* typeset	
Additional data availability information:	Tick here if the URLs/accession numbers/DOIs will be available only after acceptance of the manuscript for publication so that we can ensure their inclusion before publication.

May 24, 2024

To the esteemed editors of PLOS ONE,

I am submitting a revised copy of our manuscript entitled *"Trying to remain calm…but I do reach my limit sometimes": An exploration of the meaning of gentle parenting,* which addresses all feedback from our reviewers.

All publication and ethical guidelines were followed in the development of this manuscript. Both Dr. Davidson and I are in agreement on the content of this manuscript. This manuscript has not been published elsewhere and is not being simultaneously reviewed elsewhere for publication. We look forward to your feedback and editorial decision.

Update: Macalester College, which funded $1250 for participant compensation, had no role in the study design, data collection and analysis, decision to publish, or in preparation of the manuscript.

Sincerely,

Annie Pezalla, PhD
Macalester College
apezall1@macalester.edu

MACALESTER

Revised manuscript - clean
Click here to access/download;Manuscript;Pezalla - Davidson - PLOS - Clean.docx

1. “Trying to remain calm…but I do reach my limit sometimes”: An exploration of the meaning of gentle parenting

2. Anne E. Pezalla[a] and Alice J. Davidson[b]

3. [a]Macalester College, St. Paul, MN, United States,

4. [b]Rollins College, Winter Park, FL, United States

5. Corresponding author:

6. Email: apezall1@macalester.edu

Abstract

Raising young children has always been hard, but evidence suggests that it may be getting harder. The isolation of the pandemic, the pressures to fulfill exacting parenting standards, and the explosion of "expert" parenting advice on social media have fueled the rise of "gentle parenting," an approach that pivots away from older, discipline-heavy parenting typologies and which promises the development of happier, healthier children. Despite the popularity of gentle parenting, it has received no empirical scrutiny. The current study represents the first systematic investigation of what gentle parenting entails. Data were gathered from a sample (N = 100) of parents of at least one child between the ages of 2 and 7 from the Midwest, Southeast, and Southwest. Approximately half (n = 49) of the sample identified as "gentle parents." Inductive analyses identified this approach as one that emphasizes high levels of parental affection and parents' and children's emotion regulation. Gentle parenting appears to be distinct from other established measures of parenting approaches in its emphasis on boundaries, yet the enactment of those boundaries is not uniform. Overall, gentle parents reported high levels of parenting satisfaction and efficacy, but a subset of gentle parents who were highly critical of themselves reported significantly lower levels of efficacy than the rest of the sample. Statements of parenting uncertainty and burnout were present in over one-third of the gentle parent sample. Implications are discussed for future research and increased support for those who identify as gentle parents.

The COVID-19 pandemic left many parents around the world with a rare gift: less rushing around and more intentional time at home with their children (1). For many others, though, the pandemic was unilaterally bad. Evidence suggests that, for millions of parents, the pandemic increased parental stress (2) to levels that have not yet dropped to pre-pandemic levels (3) and lowered parents' feelings of efficacy in their parenting (4). Given the nearly impossible task of simultaneously juggling work responsibilities with caring for children at home due to the closure of schools and childcare facilities, it is no wonder that many parents of young children experienced poor mental health (5) and reported feelings of stress and exhaustion (6) during the pandemic. Those particularly vulnerable to the pandemic have been high-conflict families (1,7), families who employed harsh or inconsistent parenting (8), and families with children who were prone to stress or anxiety (9).

Evidence suggests that even beyond the pandemic, the work of rearing children is simply seen as more stressful and difficult than it was in the past. A 2023 study within the United States by the Pew Research Center (10) found that 41% of parents reported that being a parent is tiring, and 29% said it is stressful all or most of the time, with the youth mental health crises reported as a common concern among parents. Within the Pew report, 70% of mothers and 60% of fathers acknowledged that being a parent today is more demanding than it was just a few decades ago.

This spotlight on the suffering of parents is relatively new. Developmental Psychology, as a whole, has long focused on children (11), with parents "instrumentalized at the service of their children's development and best interest" (p. 8). Yet a focus on parents in their own right is merited, especially considering that *parental burnout,* which goes beyond the normality of parental stress and which severely and chronically overwhelms parents' resources to cope, is on the rise (12). Parental burnout appears to be more prevalent among parents who aim to be "perfect" (13), who are neurotic (14), and who have less social support (15). The question, then, is *what are these parents doing*?

To understand the current zeitgeist around parenting young children, it is helpful to consider the extent to which contemporary parenting approaches relate to arguably the most commonly-cited framework in parenting scholarship: Diana Baumrind's approaches. Baumrind's (16) theory-derived parent classification system, organized around the axes of parenting warmth and parenting discipline, originally generated three distinct approaches to parenting: authoritative parenting was characterized by high parental warmth and discipline; authoritarian parenting was characterized by low parental warmth and high discipline; and permissive parenting was characterized by high

parental warmth and low discipline. Maccoby and Martin (17) later expanded on Baumrind's three original parenting styles by adding a fourth type, neglectful parenting, which characterized parents who were low in both warmth and discipline. Broadly, investigations on these parenting styles highlight authoritative parenting as the optimal style for parents for its associations with wellbeing in children (18,19). Optimal parenting, according to this framework, is seen in those who employ lots of warmth and sufficient discipline and structure in their interactions with their children.

Baumrind's typology, however, has not been immune from criticism, namely, that it is Eurocentric, endorsing values that are limited to a primarily white, middle-class population (20), and that four relatively distinct parenting styles do not capture the nuance of parents' lives, since most parents inevitably model a mixture of parenting styles (21). Gottman et al. (22) critiqued the parenting styles, noting that they provide little structure to parents in specific parent-child interactions. For example, what is a parent to do when a child throws a tantrum over a toy they cannot have? In such scenarios, and in many others, Baumrind's authoritative style of high warmth and structure provides no clear guidelines. To address those limitations, Gottman and colleagues (22) proposed an alternative model that focuses on "emotion coaching," which emphasizes parents' explicit labeling of those emotions, awareness of and empathy for their child's emotions, and problem solving with their child to deal with upsetting situations. Guided by the tenets of emotion coaching, a parent whose child is in the throes of a tantrum could address the child's feelings (e.g., "you look very upset right now"), empathize with the child (e.g., "I've felt the same way as you before"), and then work on problem solving (e.g., "you can't have that toy right now but maybe after we eat dinner"). Outcomes relating to parents' use of emotion coaching have been largely positive, with emotion-coached children experiencing better self-regulation skills and less stress, although again, most of the focus has been on children (e.g., 23), and predominantly White European participants (24).

Still other parenting approaches include "positive parenting," which emphasizes consistent and unconditional empathy and guidance (25), "conscious parenting," (26), which focuses more on parents' need for introspection and mindfulness in viewing their children as teachers of important life lessons, and most recently, "gentle parenting," (27) which appears to be a catchall for variations of both "positive parenting" and "conscious parenting" and which centers on acknowledging a child's feelings and motivations behind challenging behavior, rather than correcting the behavior itself. Gentle parenting also prioritizes the notion of boundaries and giving children choices rather than orders. Gentle parenting appears to have arisen, at least in popular literature, as a

rejection of the discipline- and hierarchical-laden nature of Baumrind's authoritative parenting typology. Speculations have also been shared that gentle parenting may be the embodied rejection of how parents were themselves raised when they were children. Indeed, the recent Pew Research Center (10) report found that 44% of respondents said they wanted to raise their children differently from their own upbringing, with many of those respondents reporting that they would be more gentle and less punitive.

Further complicating the array of parenting strategies and philosophies from which contemporary parents may choose, is the fact that, unlike the above-mentioned parenting typologies, the idea of gentle parenting was not born of scholarship. Indeed, this approach seems to be fueled in great part by parenting "experts" and influencers on social media and YouTube. This reliance on social media platforms appears to have increased during the pandemic (28), and although evidence suggests that social media sources can provide helpful information about caregiving and can boost feelings of social connectedness to other parents (28,29), without discernment about the validity of parenting claims from so-called "experts," the consumption of this online content can be problematic, leading to heightened feelings of isolation and depression (30), especially among parents with higher anxiety (31). Child development researchers have not investigated the perspectives of gentle parents to determine a common definition of this approach, how this approach is related to other parenting typologies, the ways that gentle parents respond to specific challenges, and the sustainability of this approach from the perspective of the gentle parents themselves. Toward those ends, this exploratory study was guided by the following four research questions:

1. How do those who identify as "gentle parents" define such an approach?

Based on popular psychology descriptions of this new approach (27), we anticipated that "gentle parents" would have similar priorities as the "positive parenting" (25) and "conscious parenting" approaches (26), namely, emphasizing empathy and mindfulness.

2. How does "gentle parenting" relate to Baumrind's parenting typology?

Considering that "gentle parenting" seems to prioritize affection and to disavow punishment, we expected this approach to be more closely related to Baumrind's (16) authoritative and permissive parenting styles than to authoritarian or neglectful styles.

3. How do gentle parenting ideologies relate to parenting behaviors when a child "misbehaves"?
4. To what extent is a gentle parenting approach associated with parent wellbeing?

Because this is the first empirical investigation of the "gentle parenting" approach, we had no guided hypotheses for the third and fourth research questions.

Method

Participants

Participants included 100 parents of at least one young child between the ages of 2 – 7 (M_{age} = 38.58 years, *SD* = 4.07; 84 females, 16 males). Participants resided in one of three metropolitan areas in the Midwest, Southeast, and Southwest in the US. They primarily identified as White (87%), with 7% identifying as Hispanic, 5% as Asian/Pacific Islander, and 1% as Black or African American. Participants were highly educated: 65% reported a postsecondary degree; 25% had a bachelor's degree; and 10% had an associate's degree, some college, or a high school diploma or equivalent.

Procedure

The study was approved by the authors' institutional review boards (Macalester College served as the primary IRB of record [#23046]; Rollins College served as oversight IRB). These review boards were guided by the principles of The Belmont Report, federal regulations, state and local laws, and college policies. We distributed a link to a Qualtrics survey (www.qualtrics.com) via email to colleagues and personal acquaintances in various preschools, childcare facilities, and churches in the three metropolitan areas. We included a brief description of the study's goal to explore parenting practices and challenges in 2023 in the wake of the COVID-19 pandemic. Our contacts shared information about the study and the survey link with individuals in their network who met our inclusion criteria of identifying as a parent of a currently 2- to 7-year-old child. Upon opening the survey link, participants provided written informed consent after reading about the study's purpose, the risks and benefits of participation, assurances of the confidentiality of their personal information and responses, the voluntary nature of the study, and their ability to withdraw at any time. The survey took approximately 20 minutes to complete (*M* = 22.6, *SD* = 15.4) and participants received a $10 Amazon gift card as compensation within 48 hours. Data collection commenced on July 25th, 2023, and ended on October 26th, 2023, at which time we exported participants' responses to SPSS (quantitative measures) and Excel (qualitative responses) for data cleaning, coding, and analysis. Of the 111 total individuals who completed the survey, we utilized all responses for establishing qualitative coding reliability.

To ensure the credibility of responses, we included three validation questions in the survey, which asked participants to select a particular response; 11 respondents failed those questions and thus we deleted their data for our primary analyses.

Measures

Parenting Approaches

We measured parenting approaches via a list of 16 parenting descriptors and open-ended responses to a question about how they responded to their child(ren)'s misbehavior and how they approach parenting. Participants selected from a list of adjectives (accepting, affectionate, confrontational, conscious, consistent, expressive, firm, gentle, indulgent, intentional, permissive, reactive, respectful, responsive, strict, and warm) to describe how "your primary parental figure(s) approached parenting when you were a young child." They could select all that applied. Participants selected from the same list of adjectives to indicate their personal approach to parenting their 2- to 7-year-old child(ren). When they selected 'gentle,' they were prompted to describe "what does gentle mean to you as it applies to your personal approach to parenting?" Responses ranged from 3 to 77 words (*M* = 19.6, *SD* = 14.10). To assess parental responses to their child(ren)'s misbehavior, parents responded to the prompt: *Think about a recent time when your 2- to 7-year-old child was exhibiting challenging behavior (e.g., 'acting out,' misbehaving, or being disruptive at home or in public). How did you respond in that situation?* Responses ranged from 13 to 353 words (*M* = 73.91, *SD* = 70.39). Additionally, parents described their approach to parenting. Responses to this prompt ranged from 1 to 348 words (M = 47.68, SD = 59.21).

Parenting Styles

Parenting styles were measured using 19 items from the Parenting Style Scale (34), which was adapted from Baumrind's original typology. Items were scored on a 5-point Likert scale (1 = Never; 5 = Always) with 4 items measuring *indulgent* (i.e., *permissive*) *parenting* (e.g., "*I make decisions in consultation with my child.*"; α = .72), 4 items measuring *authoritative parenting* (e.g., "*I give my child reasons for my directions.*"' α = .73), 5 items measuring *authoritarian parenting* (e.g., "*I have the final say with my child.*"; α = .62) and 4 items measuring *neglectful parenting* (e.g., "*My child wins arguments with me.*"; α = .64). Items were averaged within each subscale, so individual scores could range from 1 to 5 for each parenting type. Given our interest in comparing gentle parenting to existing parenting styles, we excluded one item on the authoritative subscale that specifically described

using a "*gentle manner with my child*." For the remaining 18 items, we conducted a principal components analysis to confirm the 4 parenting styles using an oblique rotation (direct oblimin) to simplify the factors and allow factors to be correlated. The resulting structure matrix indicated four clear factors that corresponded with the intended parenting styles, with the exception of one item ("*I tell my child how happy he/she makes me*") that did not load on any of the four factors (factor score < .4). We excluded that item from the indulgent subscale.

Parent Wellbeing

Parent wellbeing was operationalized using 15 items describing *self-efficacy in the parenting role* and *satisfaction with parenting* from the Parenting Sense of Competence (PSOC) scale (35). Participants indicated their level of agreement with each item by indicating a number between 1 (strongly agree) and 6 (strongly disagree) that best corresponded to their sense of parenting self-efficacy (e.g., "*Being a parent is manageable, and any problems are easily solved*") and satisfaction (e.g., "*I go to bed the same way I wake up in the morning, feeling I have not accomplished a whole lot*"). The 8 efficacy items were reverse scored so that high scores on both subscales indicated positive parental experience. To analyze the two-factor structure of the PSOC, we conducted a principal components analysis with an oblique rotation (direct oblimin) to simplify the factors and allow factors to be correlated. The resulting structure matrix clearly indicated a two factor structure for efficacy and satisfaction. One of the satisfaction items ("My mother/father was better prepared to be a good mother/father than I am,") loaded strongly on the efficacy factor (factor score > .4), so we included the item as part of the efficacy subscale. An additional satisfaction item ("A difficult problem in being a parent is not knowing whether you're doing a good job or a bad job") did not load on either factor so we excluded it from the composite scores. Items were averaged for each subscale to create composite parenting self-efficacy (9 items) and parenting satisfaction (5 items) scores. Cronbach's alphas were adequate (α = .77 and .81 for efficacy and satisfaction, respectively) and similar to those observed with a normative sample in Gilmore and Cuskelly (36).

Coding and Data Analytic Plan

To analyze participants' open-ended descriptions of the meaning of 'gentle', we utilized an inductive approach (32), in which we first engaged in independent, close readings of the responses and open coding, making notes about any specific themes or descriptors used to illustrate what gentle parenting meant to participants. After discussing the emerging themes identified in the gentle responses, we engaged in an iterative process of (a)

developing and refining coding categories with operational definitions regarding parents' descriptions of their personal attempts to regulate their own emotions and to help their child regulate their emotions, (b) continued close readings of participants' gentle parenting descriptions, and (c) ongoing discussion about relevant examples and counterexamples in individual responses. We achieved strong inter-rater reliability for each coding category utilizing our final gentle parenting coding scheme (Cohen's $\kappa > .80$) (33).

We used a similar inductive approach for coding the ways in which parents responded to their child's misbehavior; this approach consisted of an iterative process of close readings, open coding and creation of emergent themes. We established a coding scheme consisting of 12 operationally defined parenting responses to their child's misbehavior, and engaged in several rounds of independent coding, discussion of disagreements, and refinement of the coding scheme until we achieved strong reliability (Cohen's $\kappa > .80$) for each category. We resolved all discrepancies and established the final codes for each response (see Tables 1 and 2).

Table 1. Themes in the Meanings of Gentle Parenting, According to Gentle Parents (*n* = 49).

Coding Category (Inter-rater reliability; Frequency of responses; Percentage of responses)	Operational Definition	Example
Parent regulates own emotions		
Staying calm (κ = .96; N = 23; 47%)	Parent emphasizes their maintenance of a calm state, such as keeping voice at a low, soft tone; taking deep breaths; taking time to respond; no yelling, screaming, or threats	Having a moderate reaction - never getting too alarmed or being too permissive, always monitoring and adjusting expectations to the needs of the child and environment.
No physical punishment or punitive measures (κ = 1.00; N = 10; 20%)	Parent explicitly states that they refrain from hitting, spanking, or grabbing their child.	To me gentle refers to my response in frustrating or highly emotional situations with our daughter. We do not hit, spank, demean, or punish our child to make them feel like less of a person. Gentle refers to our discipline techniques and how we respect our child enough to make better decisions on her own. Even if we have to say something many times, we still are not aggressive towards our child to get the point across.
Parent helps child regulate their emotions		
Emotion validation (κ = 1.00; N = 15; 31%)	Parent attends to, respects, tries to understand, and validates child's feelings/needs. This includes listening with empathy.	Gentle means my children feel that they can come to me with an issue or concern and be received with patience and feel heard.
Emotion scaffolding (κ = 1.00; N = 7; 14%)	Parent uses child's emotional experiences as teachable moments for growth and self-understanding. This includes helping child learn coping skills, such as labeling emotions with words, and helping child "get to the root of her feelings." This also includes "processing" with children, and using clear communication with a rationale provided, or explaining the 'why' behind decisions.	Always providing a soft landing pad for him to explore his own thoughts and processes…gentle to me really means that he knows that I will consistently guide him without anger or frustration directed towards him as he learns to navigate the world and his place in it.
Parent shows affection (κ = 1.00; N = 13; 27%)	Parent shows loving emotional and physical affection to child (e.g., hugs); descriptions of being loving, kind, warm	Soft language, maybe a hug & sitting together for awhile

Table 2. Themes in Gentle Parents' (*n* = 49) Narratives of Response to Child's Misbehavior.

Coding Category (Inter-rater reliability; frequency; percentage)	Definition	Example
PARENT-DIRECTED RESPONSES		
Punish (κ = .96; N = 15; 31%)	Either taking away a privilege (e.g., screen time or treats) or administering unpleasant stimuli (e.g., yelling, grabbing their arm, forcing an apology) in response to misbehavior. Allowing a natural consequence that is unpleasant for the child.	I tell them to knock it off.<br><br>[I] separate and punish them,
Call them out (κ = 1.00; N = 15; 31%)	Telling child that their behavior is not appropriate. Directly addresses the misbehavior.	I tell them that that behavior is unacceptable.<br><br>I said firmly that her behavior wasn't okay. (Something like "This behavior needs to stop. It's hurtful and not helpful.")
Rationalize (κ = .96; N = 24; 49%)	Explaining the reasons why their behavior was not okay. This reasoning can occur during misbehavior and sometimes afterwards when processing/debriefing the incident.	Try to explain the circumstances ("although I read that toddlers can't rationalize so well").<br><br>I explain why changing his behavior was important. I tried to explain it in a way that reflected the importance in relation to him and those around him.
Redirect (κ = 1.00; N = 10; 20%)	Attempting to re-channel children's 'acting out' or misbehavior to something more productive/healthy; offering a positive alternative to misbehavior that does not involve bribery.	I tried to redirect to do something positive with her like have some cuddle time and read a book together.<br><br>I offer and model a replacement behavior.
Disengage (κ = .85; N = 3; 6%)	Explicitly stepping back from or leaving an emotionally intense scenario in an effort to stay calm. Usually manifested in walking away and/or taking a deep breath. Stating the need to "hold/honor a boundary" around their own wellbeing and prioritizing that boundary over their child's demands.	I had to walk away and disengage to send the message that the extreme behavior will not get a response.<br><br>I usually try to take a second to compose myself

CHILD-CENTERED OR CO-CONSTRUCTED RESPONSES

Show affection<br>(κ = 1.00; N = 10; 20%)	Providing any physical affection (e.g., hugging) or emotional affection (e.g., "I tell her I love her").	Whenever there is a set-back in this process, we say “do you need a hug?<br><br>Reminded her I loved her.
Emphasize calm<br>(κ = .96; N = 14; 26%)	Trying to encourage/assist their child in calming their bodies when they are upset. Working towards a calm goal-state in the child.	I usually try to help my child regain her calm (to regulate, suggest some deep breathing or naming the big emotion) and have her use more appropriate behaviors (calm tone or “tell me in words please so I may understand you” type of directives) to come up with a choice/resolution.
Let it fly<br>(κ = 1.00; N = 19; 39%)	Letting the child “feel their feelings” without interruption or interference.	Waited until his fit was over.<br><br>I let them have their feelings about it.
Minimize hierarchy<br>(κ = 1.00; N = 12; 25%)	Attempting to “get on child’s level”, either physically (e.g., bringing body at the same height of child, sitting with them) or emotionally (e.g., acknowledging feelings, validating feelings).	Sat my child down on a nearby chair, knelt down so we were on the same level, made physical contact & eye contact<br><br>I validated their experience
Bargain<br>(κ = 1.00; N = 5; 10%)	Negotiating with or bribing the child (e.g., giving food or screens) under the assumption that their “misbehavior” stemmed from an unmet need. This can also be preemptive bribes to help elicit desired behavior.	Asked them what they needed in the moment, tried to provide that if it was appropriate to the situation.
Ask<br>(κ = 1.00; N = 5; 10%)	Asking child to stop misbehaving (e.g., can you use your words?).	Typically we ask several times to stop.<br><br>Asked nicely for them to please stop said behavior (being mean to little brother, not listening when I ask him to do something, etc.)

In addition, we examined specific instances of parenting approach descriptions to capture a more holistic picture of the philosophy and practice informing gentle parents' responses to their child's misbehavior and to better understand gentle parents' wellbeing after conducting our primary analyses (See Results).

To address our four research questions, our data analysis involved a mix of descriptive statistics (frequencies of identified qualitative themes regarding the meaning of 'gentle', adjectives used to describe personal v. parents' approaches to parenting, and misbehavior responses) and independent samples *t*-tests to compare gentle v. not-gentle parents on parenting styles and wellbeing. To control for Type I error due to multiple comparisons, we applied a Bonferroni correction for nine pairwise comparisons: age, number of children, four parenting styles (authoritative, authoritarian, permissive, neglectful), parenting efficacy, parenting satisfaction, and total adjectives. Given these nine comparisons, and our experiment-wise alpha level of 0.05, the corrected alpha was set to .006 ($p < 0.05/9$).

Results

How do those who identify as "gentle parents" define such an approach?

To explore our first research question, we identified the primary themes that emerged in the definitions of gentle parenting reported by the 49 participants who selected "gentle" as an adjective to describe their personal approach to parenting. The most prominent theme involved parents' descriptions of regulating their own emotions (59.2%), which emphasized staying calm and/or avoiding physical punishment or any punitive measures with their child (see Table 1). For example, one gentle parent, a 42-year-old mother of two children, ages 7 months and 3 years, noted:

> To me, gentle refers to my response in frustrating or highly emotional situations with our daughter. We do not hit, spank, demean, or punish our child to make them feel like less of a person. Gentle refers to our discipline techniques and how we respect our child enough to make better decisions on her own. Even if we have to say something many times, we still are not aggressive towards our child to get the point across.

A secondary theme highlighted parents' descriptions of assisting their children in regulating their emotions (40.8%), which involved validating and listening to children's emotional experiences and/or scaffolding emotions, such that they used their child's emotional experiences as teachable moments for growth and self-understanding. This theme was evident in the response of another gentle parent, a 36-year-old mother of two children, ages 1 and 3, who described the approach as "helping my kids understand how their feelings can impact themselves and those around them so that they can build healthy coping skills." Finally, a third theme captured parents' descriptions of showing physical and/or emotional affection to their child (26.5%), such as one parent, a 38-year-old mother of three children, ages 3, 6, and 8, who commented that to her, gentle parenting involves "showing them affection that shows I care and love them."

Gentle parenting definitions were often multi-thematic. Thirty-four percent (n = 17) of the descriptions of gentle parenting in our sample referenced more than one emergent theme, such as this response from a 37-year-old father of three children, ages 2, 5, and 7 [with **bolded inserts** added to illuminate themes]: "Gentle parenting means mostly maintaining a lower energy level [**parent regulates own emotions]** and being respectful of my children's need for rest and relaxation [**parent regulates child's emotions]**", or this response, from a 39-year-old mother of two children, ages 3 and 5: "Gentle parenting means no harsh words [**parent regulates own emotions]** and soft touches [**shows affection].**" Responses that defined gentle parenting with only one theme (n = 32) were still richly descriptive (average word count of single-themed responses was 20 words), such as the following response:

> To me, gentle refers to my response [**parent regulates own emotions]** in frustrating or highly emotional situations with our daughter. We do not hit, spank, demean, or punish our child to make them feel like less of a person. Gentle refers to our discipline techniques and how we respect our child enough to make better decisions on her own. Even if we have to say something many times, we still are not aggressive towards our child to get the point across. - *42-year-old mother of two children, ages 6 and 10.*

To further explore the meaning of 'gentle' to self-identified gentle parents, we analyzed additional descriptors participants indicated regarding their approach to parenting. When participants selected gentle to describe their own parenting, 94% also selected affectionate, 88% selected accepting, 86% selected warm, 82% intentional, 78% respectful, and 71% selected conscious. This exploration also involved a series of Pearson Chi-Square tests to compare gentle and non-gentle parents in terms of the frequency with which they selected specific adjectives (other than gentle) to describe their personal approach to parenting. Gentle parents were statistically more

likely to describe themselves as ‘expressive’ (*rf* = .57) than non-gentle parents were (*rf* = .35), X^2(1, N = 100) = 4.80, *p* = .028, but they did not differ from non-gentle parents in the frequency with which they selected any other adjectives to describe their approach to parenting. We also compared gentle and non-gentle parents on the total number of descriptors they used to describe their parenting approach. An independent samples *t*-test indicated that gentle parents selected significantly more adjectives overall to describe their own parenting (*M* = 8.65, *SD* = 2.06) in comparison to non-gentle parents (*M* = 6.94, *SD* = 1.83), *t*(98) = 4.41, *p* < .001. A paired samples *t*-test indicated that gentle parents also provided approximately 50% more adjectives to describe their own parenting approach in comparison to the number of adjectives they used to describe their own parents (*M* = 5.98, *SD* = 2.23), *t*(48) = 9.70, *p* < .001), describing themselves as being more accepting, affectionate, conscious, consistent, expressive, intentional, respectful, and warm, and less indulgent, reactive, and strict (see Figure 1). Importantly, these differences were not unique to gentle parents: non-gentle parents also described their own parenting style with more adjectives in comparison to the number of adjectives they used to describe how they were reared by their parents (*M* = 4.94, *SD* = 2.00), *t*(50) = 7.32, *p* < .001.

Figure 1. How do you describe your parenting approach vs. how did your parents raise you? Differences in adjectives among gentle parents (*n* = 49).

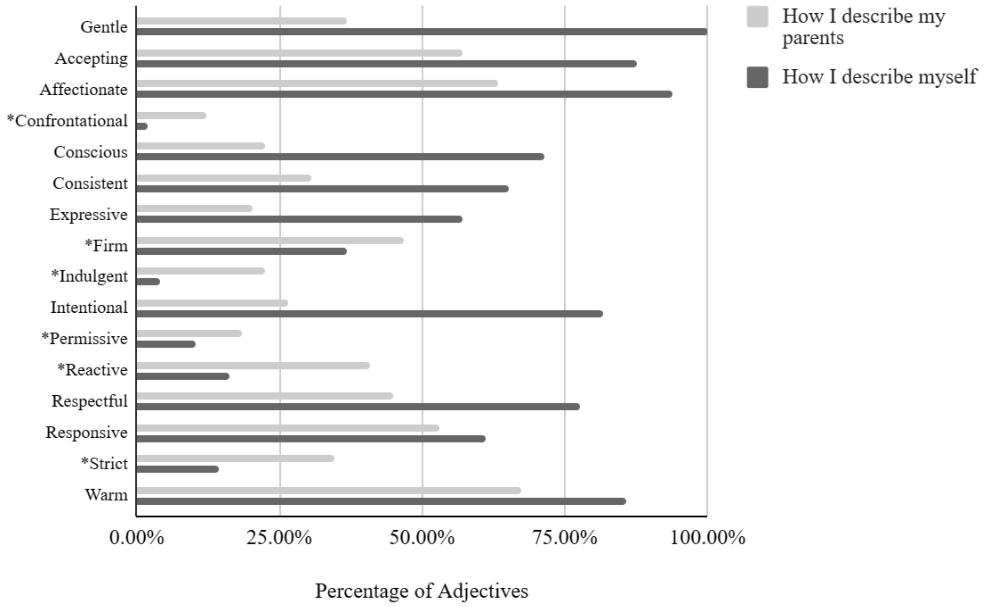

Note.* indicates a statistically significant (*p* < .05) difference in the percentage of adjectives that gentle parents (*n* = 49) used to describe their own parenting approach versus the number of adjectives used to describe how they were raised by their parents.

How does gentle parenting relate to Baumrind's parenting typology?

To examine our second research question, we conducted a series of independent samples *t*-tests to compare differences in authoritative, authoritarian, indulgent, and neglectful parenting between gentle and non-gentle parents. Compared to those who did not identify as gentle in their parenting approach, gentle parents had higher scores on authoritative and indulgent parenting, and lower scores on authoritarian and neglectful parenting, though these differences did not reach statistical significance with the Bonferroni corrections ($p < .006$) (see Table 3).

Table 3. Differences Between Gentle and Non-Gentle Parents in Baumrind's Parenting Approaches, Age, and Number of Children.

Variable	Gentle Parents (*n* = 49)		Not Gentle (*n* = 51)		*t*(98)	*p*	Cohen's *d*
Parenting Approach	*M*	*SD*	*M*	*SD*			
Indulgent	4.20	0.47	4.03	0.53	1.65	.051	.186
Authoritative	3.95	0.45	3.85	0.61	.745	.458	.152
Authoritarian	3.38	0.50	3.39	0.41	-.504	.615	.102
Neglectful	2.40	0.46	2.41	0.48	-.131	.896	.027
Age	39.02	3.83	38.16	4.27	1.063	.291	.213
Number of Children	2.10	0.77	2.12	0.65	.109	.913	.022

How do gentle parenting ideologies relate to parenting behaviors when a child "misbehaves"?

To explore the ways in which gentle parents responded to instances of their child(ren)'s misbehavior, we first calculated the frequency with which parents employed each of the 12 parenting responses (see Table 2). Similar to the definitions of gentle parenting, the responses to misbehavior were multifaceted, often illustrating a number of approaches. On average, gentle parents employed three different responses in these moments of misbehavior from their children (*M* = 2.96, *SD* = 1.33), with rationalizing (47%) and letting it fly (36%) as the most common responses. The excerpt below illuminates this multifaceted response to misbehavior [**bolded inserts** added to illuminate the tagged code]:

> I have noticed that my responses differ in the situation (likely not good). At home - we take quite a bit of time to get low to the ground (at her level)], affirm her emotions ("I see you are upset right now") [**minimize hierarchy**], try to understand the cause (if it's not obvious), try to explain the circumstances (although I read that toddlers can't rationalize so well) [**rationalize**], and sometimes try to let her solve the problem for herself (probably less often), offer acceptable choices for moving forward that result in an acceptable outcome for us ("do you want to do x this way or that way") **[redirect]**. Whenever there is a set-back in this process, we say "do you need a hug? Let's hug it out." **[show affection]** then proceed to the next step. - *42-year-old mother of one child, age 3.*

To further explore those responses to children's misbehavior, we categorized the 12 distinct parenting responses into what we perceived to be either parent-directed responses or child-directed responses. Parent-directed responses were those that were guided by the parent and that represented firmer *boundaries,* or demarcated rules and/or limits, between the parent and child. These responses included punishing, calling them out, rationalizing, redirecting, and disengaging. Child-directed responses were those that were more child-centered and prioritized giving the child more control, power, and/or attention; they also de-emphasized the importance of or minimized the boundaries between the parent and child, reflecting a more democratic parenting approach. These responses included showing affection, emphasizing calm, letting it fly, asking, minimizing hierarchy, and bargaining. Within each of the misbehavior narratives, participants received a binary score of either 0 or 1 for each of the 12 parenting responses, depending on whether that response was evident in the narrative. We then summed the total number of

parent-directed responses and the total number of child-directed responses for each participant. Overall, gentle parents were equally likely to describe parent-directed (M = 1.37, SD = .88) and child-directed (M = 1.33, SD = 1.07) responses, $t(48)$ = .185, p = .854. There was, however, much variation among parents within these two categories.

To capture variation in the extent to which gentle parents favored a parent-directed or child-directed approach, we computed a difference score, subtracting the number of child-directed responses from the number of parent-directed responses, such that positive scores indicated participants' inclusion of more parent-directed responses in their misbehavior narratives, negative scores indicated more child-directed responses, and scores of 0 indicated that self-identified gentle parents described an equal mix of parent- and child-directed responses. Consistent with findings that gentle parenting was multi-thematic and multifaceted, there was variation in gentle parents' difference scores indicating that gentle parenting is practiced on a spectrum. Difference scores ranged from -3 (higher on child-directed misbehavior responses) to 3 (higher on parent-directed responses). Most gentle parents (67%) described a combination of parent-directed and child-directed responses. Twenty gentle parents had negative difference scores indicating misbehavior response descriptions that included more child-directed responses, 18 gentle parents had positive difference scores, indicating descriptions with more parent-directed responses, and 11 gentle parents had scores of 0 indicating an equal number of parent- and child-directed responses described in their misbehavior narratives.

The following narrative illustrates a misbehavior response from a parent who described using only child-directed strategies (difference score = -3) [**bolded inserts** added to illuminate the tagged child-directed code]:

> We try our best to stay calm when children are "acting out." We let them feel their feelings [**let it fly**], and if they're really upset, go with them to another room to calm their bodies down [**emphasize calm**]. Later in the day when they are calmed down, we talk through what happened and why they felt that way [**minimize hierarchy**], what they could do instead if they got physical. Sometimes we don't react the way we want as parents! We never yell, but are sometimes more firm or say things we didn't mean to say. In those situations, we apologize and talk. *-35-year-old mother of two children, ages 1 and 4.*

Throughout this mother's response, the emphasis is not on parent-governed rules or expectations, but rather, the focus is on the child's feelings, needs, and abilities to (later) process whatever misbehavior had occurred. Parent-directed boundaries are not emphasized in these child-directed responses.

Contrasting those child-directed strategies were those from parents who endorsed a parent-directed response to misbehavior, such as the following (difference score = + 3) [**bolded inserts** added to illuminate the tagged parent-directed code]:

> Last night, she was in the bathtub and kicking the water so that it was splashing out of the tub. I explained to her that she could kick smaller in the tub, but big kicks are for the pool [**call them out**]. Big kicks in the tub is getting water everywhere. I explained that if she kept splashing, bathtime would be over [**rationalize**]. She was laughing and continued to kick water out of the tub, so I started draining the water, picked her out of the tub, and gave her the towel [**punish**]. She got back in the tub, and I repeated, no, bathtime is over, picked her out of the tub, and put the towel around her. She got upset and was crying. I think I acknowledged that it's hard, but that she needs to do little kicks in the tub. *- 37-year-old mother of two children, ages 1 and 3.*

Throughout this mother's response, the emphasis is on boundaries–on clearly stated rules and/or limits, set and maintained by the parent–even when there was clear disagreement about those rules from the child.

Situated between those two distinct approaches to misbehavior were the gentle parents who equally referenced a combination of parent- and child-directed responses when they discussed how they responded to their child(ren)'s misbehavior. The following response generated a difference score of zero, since it included both punishing (a parent-directed response) and bargaining (a child-directed response) [**bolded inserts** added to illuminate the tagged parent- and child-directed code]:

> [I] Tell the child how to act, walking feet, inside voices. Since my child is young if we go somewhere I feel a behavior might happen I remind them of expected behavior at that location and if they do so what they will earn [**bargain]**. When they don't have expected behavior they don't get to earn privileges [**punish]**.
> *- 33-year-old mother of one child, age 3.*

To what extent is a gentle parenting approach associated with parent wellbeing?

In the current study, we had no directional hypotheses about whether gentle parents would differ from non-gentle parents in terms of their parenting self-efficacy or satisfaction. To explore this potential variance, we conducted two-tailed, independent-samples *t*-tests to determine the extent to which mean differences in parenting

efficacy and parenting satisfaction differed among those who identified as a gentle parent versus those who did not. Parenting self-efficacy did not differ significantly among gentle- versus non-gentle parents ($t(98) = 1.48$, $p = .142$), although mean scores were higher among gentle- ($M = 4.32$, $SD = 0.61$) compared to non-gentle parents ($M = 4.13$, $SD = 0.65$). Gentle parents reported more satisfaction ($M = 4.15$, $SD = 0.96$) than did non-gentle parents ($M = 3.73$, $SD = 0.92$), although the difference did not reach statistical significance with the Bonferroni correction, $t(98) = 2.26$, $p = 0.026$.

In slight contrast to these quantitative findings was a theme that arose from the qualitative data. Within the narratives gentle parents provided, they often reflected on their wellbeing, openly questioning whether they were "doing it [parenting] right." To gain a better understanding of these self-evaluations, we studied the parenting approach descriptions parents provided and we identified additional, similar, evaluative reflections. In response to the question about parenting approaches, one gentle parent, a 38-year-old mother of three children, wrote simply: "I'm hanging on for dear life." Another, a 43-year-old mother of two children, ages 3 and 5, wrote that "I confess I feel I have no idea what I'm doing much of the time." Following this emergent theme, we read across all gentle parents' narrative responses to identify any utterances of what we called *self-critique,* which we defined as moments of parenting uncertainty, insecurity, and/or doubts about personal parenting efforts/attempts. Overall, 34.7% of gentle parents' responses about their parenting approach or about how they handled their children's misbehavior contained unprompted elicitations of self-critique. Below are several examples of gentle parents' self-critique about their parenting approach or response to their child's misbehavior:

> I would describe it [my parenting approach] as still exploring. I have read ALL the parenting books, listened to the podcasts, followed the socials… and can't say I feel I've found any one "philosophy" that we adhere to all the time…I don't really feel like I know what I'm doing - so still exploring what "my approach" is. *-38-year-old mother of two children, ages 5 and 7.*

> As a stay at home mother I get easily overstimulated and overwhelmed all day every day. I very much look forward to nap time and bedtime because those are pretty much the only times I get to be alone. *-36-year-old mother of two children, ages 1 and 4.*

> In public…we tend to rush through the normal process, skip steps, with varying degrees of success. When/if they don't work, we are more likely to transition into bribing/negotiating - which we are aware are not recommended approaches. We have observed that we use food too often as the carrot, which we need to work on. - *42-year-old mother of one child, age 3.*

Because of the prevalence of the self-critique theme in gentle parents' responses, we explored the extent to which parenting efficacy and parenting satisfaction differed among those gentle parents who self-critiqued versus those who did not. Among all gentle parents in our sample, parenting self-efficacy among those who engaged in self-critique (M = 3.96, SD = 0.46) was significantly lower ($t(47) = 2.69$, $p = .01$) than the parenting self-efficacy of those who did not engage in self-critique (M = 4.44, SD = 0.65). Parenting satisfaction among those who engaged in self-critique (M = 3.94, SD = 0.90) also was lower than the parenting satisfaction of those who did not engage in self-critique (M = 4.13, SD = 0.70), but this difference was not statistically significant, $t(47) = 0.79$, $p = .434$)

Discussion

Parenting young children has always been hard, but evidence suggests that it might be getting harder (10). The pressures to fulfill exacting parenting standards, coupled with the information overload on social media about the right or wrong ways to care for children, has left many parents questioning their moment-to-moment interactions with their family and leaving them with feelings of burnout (2). Such feelings may have emerged as many caregivers increased their time spent in active parenting (i.e., more play/learning activities) and decreased their time spent disciplining their young children during the lockdown period, a trend that has, notably, emerged across the income spectrum (1). Within this time of parenting angst, the idea of "gentle parenting" has gained popularity, especially on social media, as a promising new approach to raise happier, healthier children. Since the idea of gentle parenting was first introduced in 2015 by British author and parent Sarah Ockwell-Smith, coverage of this approach has skyrocketed online. At the time of this publication, a Google search on "gentle parenting" generates 1.68 million hits. That number will undoubtedly continue to climb.

Despite the popularity of this approach, there has been no empirical research about what gentle parenting actually means among those who endorse this approach; no systematic investigations to interrogate the extent to which gentle parenting is, in fact, a distinct approach from other, established typologies of parenting; no explorations to determine how gentle parents respond to challenging moments with their children; and equally

important, no pointed inquiries to consider whether gentle parents are faring any better than the rest of us. This study represents a preliminary step to address those important questions.

The meaning of 'gentle parenting' according to gentle parents

Forty-nine percent of our sample described their personal approach to parenting as 'gentle'. These gentle parents ranged from 32 to 51 years of age, and they were no more or less likely to have additional children than non-gentle parents. At least among the three US urban populations from which we sampled, gentle parenting appears to be a widely endorsed approach to parenting young children among millennials and Gen-Xers. Our analyses suggest that gentle parenting is nuanced and multi-thematic, especially in comparison to the ways these gentle parents were raised when they were children, but it is primarily an approach that prioritizes parents' and children's emotion regulation and a high amount of parental affection. Almost 60% of self-identified gentle parents defined 'gentle' as regulating their own emotions, which involved maintaining a sense of calmness and a cool head, as well as avoiding any punitive actions, such as yelling or spanking, when it came to parenting their young children. Consistent with our expectations, this theme is similar to contemporary positive parenting movements that emphasize the importance of caregivers remaining 'mindful' (25) and 'conscious' (26). Parent emotion expression and regulation have long been considered integral to young children's developing social and emotional competence (37). Parents provide the primary context in which children first learn about healthy (and unhealthy) emotion expression and management (38), and evidence suggests that parents with better emotion regulation skills exhibit more positive parenting behaviors and have children with better emotion regulation and fewer internalizing symptoms (39). Close to 41% of the self-identified gentle parents in our sample also defined 'gentle' as assisting their children with emotion regulation. These parents emphasized the importance of validating their child's emotions through listening with empathy, understanding, and respect, as well as using their child's emotional experiences as opportunities for nurturing intimacy and teaching coping skills and problem-solving. Both of these themes are consistent with Gottman and colleagues' (22) model of emotion coaching, which affirms the role of caregivers' empathetic engagement and awareness of emotions in themselves, as well their critical role in acknowledging and scaffolding their children's emotional expressions. A third theme mentioned by around a quarter of gentle parents focused on showing physical and/or emotional affection to their child. These descriptions of hugging their child and being loving, kind, and warm are consistent with what positive parenting endorses (e.g., 40), and such maternal and paternal behaviors are largely positive in terms of their correlates with child outcomes (41,42).

Associations between gentle parenting and Baumrind's typology

As to whether or not gentle parenting is truly a unique approach or is, figuratively, "old wine in new skins," the evidence from this study suggests that, consistent with our expectations, gentle parenting is similar to Baumrind's (1966) authoritative and indulgent parenting styles; based on the adjectives gentle parents used to describe their approach, it also seems to be similar to the positive parenting model (40,41). Still, this approach likely merits its own qualitatively distinct category in that it emphasizes *boundaries* as a novel alternative to discipline. In our analyses of gentle parents' responses to their children's misbehavior, we noted that 28% included a reference to boundaries. "We try to hold consistent boundaries," wrote a 39-year-old mother of two kids, ages 3 and 11. "We focus a lot on communicating boundaries," wrote a 35-year-old father of one child, age 5. This emphasis on boundaries is distinct from other parenting approaches, but interestingly, gentle parents' behaviors–or at least, their self-reports of their behaviors–showed a great deal of diversity in terms of how those boundaries were carried out in moments of their children's misbehavior. About 40% of gentle parents in our sample demonstrated fairly clear, demarcated rules or boundaries with their children (what we referred to as a parent-directed approach); about 40% illustrated a more child-directed, democratic approach, with less-clear boundaries; and the remainder showed a combination of approaches, some that minimized boundaries and others that bolstered them. This diversity underscores the notion that gentle parenting is not a uniformly applied approach, but rather a multi-thematic philosophy and a multifaceted practice that is best conceptualized on a spectrum.

"I do the opposite of my parents"

A common sentiment expressed among the gentle parents in our sample was that they would do a better job at rearing their young children than their own parents had done with them. In comparison to their descriptions of their own parents, gentle parents described themselves as more accepting, affectionate, conscious, expressive, respectful, responsive, warm, and intentional. Indeed, "more, more, more" was the theme of these gentle parents. On average, they reported 50% more adjectives in describing their own parenting style than they used to describe how they were raised by their parents. These gentle parents would do more for their children. They would *be* more.

For some in our sample, the emphasis seemed to be about undoing what they perceived to be harmful parenting practices that they had experienced when they were children. For example, one gentle parent, a 43-year-old mother of three children, ages 8, 4, and 8 months, wrote that her approach to parenting was, quite simply, to "do

the opposite of my parents. No spanking or physical punishment." This desire to not only "do more" for their children but to "do differently" can be seen in the adjectives gentle parents selected to describe themselves versus their parents. Eighty-two percent of gentle parents in our sample described themselves as "intentional," a stark contrast from the "reactive" descriptor that 42% of them ascribed to their parents. These findings are aligned with the recent report from the Pew Research Center (10), which revealed that 43% of the sampled parents wanted to raise their children differently than they had been raised, specifically with more love and compassion. This [perception of an] inter-generational divide in parenting values is meaningful, and represents an under-explored area of research in developmental psychology. Future research should examine the relationships between self-identified gentle parents and their own parents to further elucidate the findings in our study that a subset of gentle parents were rejecting the reactive manner in which they were parented.

Gentle parents are not-so-gentle on themselves

As to how these gentle parents are doing, our data, again, portray a nuanced portrait. Overall, the gentle parents in this study reported high levels of parenting self-efficacy and satisfaction, suggesting that on the whole, they are doing quite well. But the emergent theme of self-critique, expressed by over one-third of gentle parents, and the findings that, among those self-critical gentle parents, the levels of self-efficacy were significantly lower, illuminates the need for more explorations and more support of these parents. One of the gentle parents in our sample, a 40-year-old mother of two children, wrote that her approach to parenting is about "Trying to remain calm…but I do reach my limit sometimes." Gentle parenting seems to represent an approach that is extraordinarily gentle for the children, but perhaps not-so-gentle for the parents themselves. Future research would benefit from examining gentle parenting in association with additional measures of parent wellbeing, including parenting stress and perfectionism.

Limitations and future research directions

The limitations of this study include the relatively small, homogeneous sample. We wonder: Is gentle parenting a predominantly White, high socioeconomic status approach to parenting among mothers? Is gentle parenting a stressful experience (at least for some) because of its ideologies, or because of other, unexplored variables? Future research is merited with a more diverse sample, one that accounts for both parents' and child(ren)'s age and gender, the number of children in the family, and family structure, to explore the extent to

which gentle parenting is defined and/or practiced similarly among other demographic groups. The current study also was limited in its means of data collection. Although our survey data were rich in detail, it was limited in that it only gathered parents' self-reports on their "general" responses, rather than "child-specific" responses; this approach may not reflect parents' authentic behaviors with their child(ren), and it may have masked important variability among parents who behave in specific ways with one child versus another. Observational data of parent-child interactions are needed, ideally in naturalistic settings. Finally, the current study was limited in its single-time-point assessment of gentle parenting. Especially considering that many gentle parents in this study were self-critical–questioning the merits of this approach and even "overwhelmed all day every day"--longitudinal research is needed on the sustainability of this approach for the parents, as well as its potential impact on child development.

Conclusions and practical implications

Guidance about the "best" ways to rear small children has been around for a long time. What seems to be unique about the gentle parenting movement is that it has not been presented or advocated by scholars of human development; rather, it has largely been the product of social media. Considering that parents are increasingly stressed or burned out by their caregiving responsibilities (12), it is imperative that evidence-based guidance is made available to those who are interested in gentle parenting. What does this approach entail? How is it related to other parenting approaches? Is it a sustainable approach for caregivers? These are empirical questions, and they deserve empirical answers. The current study represents the first systematic investigation of a parenting approach widely endorsed by contemporary parents of young children.

References

1. Schmeer KK, Singletary B, Purtell KM, Justice LM. Family disruption and parenting during the COVID-19 pandemic. Journal of Family Issues. 2023 Jan;44(1):112-38.
2. Desimpelaere EN, Soenens B, Prinzie P, Waterschoot J, Vansteenkiste M, Morbée S, Schrooyen C, De Pauw SS. Parents' Stress, Parental Burnout, and Parenting Behavior during the COVID-19 Pandemic: Comparing Parents of Children with and without Complex Care Needs. Journal of Child and Family Studies. 2023 Dec;32(12):3681-96.
3. Adams EL, Smith D, Caccavale LJ, Bean MK. Parents are stressed! Patterns of parent stress across COVID-19. Frontiers in psychiatry. 2021 Apr 8;12:626456.
4. Xue A, Oros V, La Marca-Ghaemmaghami P, Scholkmann F, Righini-Grunder F, Natalucci G, Karen T, Bassler D, Restin T. New parents experienced lower parenting self-efficacy during the COVID-19 pandemic lockdown. Children. 2021 Jan 24;8(2):79.
5. Kerr ML, Rasmussen HF, Fanning KA, Braaten SM. Parenting during COVID-19: A study of parents' experiences across gender and income levels. Family Relations. 2021 Dec;70(5):1327-42.
6. Dawes J, May T, McKinlay A, Fancourt D, Burton A. Impact of the COVID-19 pandemic on the mental health and wellbeing of parents with young children: a qualitative interview study. BMC psychology. 2021 Dec;9(1):1-3.
7. Low A, Yu Y, Sim LW, Bureau JF, Tan NC, Chen H, Yang Y, Cheon B, Lee K, Bakermans-Kranenburg M, Tsotsi S. Maternal distress and parenting during COVID-19: differential effects related to pre-pandemic distress?. BMC psychiatry. 2023 May 29;23(1):374.
8. Ostrov JM, Murray-Close D, Perry KJ, Perhamus GR, Memba GV, Rice DR, Nowalis S. Parenting and Adjustment Problems among Preschoolers during COVID-19. Journal of child and family studies. 2023 Jan;32(1):93-109.
9. Tatsiopoulou P, Holeva V, Nikopoulou VA, Parlapani E, Diakogiannis I. Children's anxiety and parenting self-efficacy during the COVID-19-related home confinement. Child: Care, Health and Development. 2022 Nov;48(6):1103-11.
10. Minkin R, Horowitz JM. Parenting in America Today.

11. Mikolajczak M, Roskam I. Parental burnout: Moving the focus from children to parents. New directions for child and adolescent development. 2020 Nov;2020(174):7-13.

12. Mikolajczak M, Gross JJ, Roskam I. Parental burnout: What is it, and why does it matter?. Clinical Psychological Science. 2019 Nov;7(6):1319-29.

13. Sorkkila M, Aunola K. Risk factors for parental burnout among Finnish parents: The role of socially prescribed perfectionism. Journal of Child and Family Studies. 2020 Mar;29:648-59.

14. Lebert-Charron A, Dorard G, Boujut E, Wendland J. Maternal burnout syndrome: Contextual and psychological associated factors. Frontiers in psychology. 2018 Jun 5;9:885.

15. Séjourné N, Sanchez-Rodriguez R, Leboullenger A, Callahan S. Maternal burn-out: an exploratory study. Journal of reproductive and infant psychology. 2018 May 27;36(3):276-88.

16. Baumrind D. Effects of authoritative parental control on child behavior. Child development. 1966 Dec 1:887-907.

17. Maccoby EE, Martin JA. Socialization in the Context of Family: Parent-Child Interaction. W: PH Munsen (ed. tomu), EM Hetherington (ed. serii), Handbook of Child Psychology, t. 4, 1–101.

18. Baumrind D. The influence of parenting style on adolescent competence and substance use. The journal of early adolescence. 1991 Feb;11(1):56-95.

19. Pinquart M, Gerke DC. Associations of parenting styles with self-esteem in children and adolescents: A meta-analysis. Journal of Child and Family Studies. 2019 Aug 5;28:2017-35.

20. Sorkhabi N, Mandara J. Are the effects of Baumrind's parenting styles culturally specific or culturally equivalent?.

21. Kuppens S, Ceulemans E. Parenting styles: A closer look at a well-known concept. Journal of child and family studies. 2019 Jan 15;28:168-81.

22. Gottman JM, Katz LF, Hooven C. Parental meta-emotion philosophy and the emotional life of families: theoretical models and preliminary data. Journal of family psychology. 1996 Sep;10(3):243.

23. Dunsmore JC, Booker JA, Ollendick TH, Greene RW. Emotion socialization in the context of risk and psychopathology: Maternal emotion coaching predicts better treatment outcomes for emotionally labile children with oppositional defiant disorder. Social Development. 2016 Feb;25(1):8-26.

24. Dudeney J, Hawes D, Johnson A, Eisenberg N, Kohlhoff J. Emotion socialization and child conduct problems: A comprehensive review and meta-analysis.
25. Rodrigo MJ, Almeida A, Spiel C, Koops W. Introduction: Evidence-based parent education programmes to promote positive parenting. European Journal of Developmental Psychology. 2012 Jan 1;9(1):2-10.
26. Tsabary S. The conscious parent: Transforming ourselves, empowering our children. Hachette UK; 2014 Aug 28.
27. Ockwell-Smith S. The gentle parenting book: How to raise calmer, happier children from birth to seven. Piatkus; 2016 Mar 3.
28. Anderson M, Vogels EA. Americans turn to technology during COVID-19 outbreak, say an outage would be a problem.
29. Hooper A, Schweiker C, Kerch C. Social support in a parenting Facebook group during the COVID-19 pandemic. Family Relations. 2023 Apr;72(2):530-46.
30. Newsham G, Drouin M, McDaniel BT. Problematic phone use, depression, and technology interference among mothers. Psychology of popular media. 2020 Apr;9(2):117.
31. Drouin M, McDaniel BT, Pater J, Toscos T. How parents and their children used social media and technology at the beginning of the COVID-19 pandemic and associations with anxiety. Cyberpsychology, Behavior, and Social Networking. 2020 Nov 1;23(11):727-36.
32. Strauss A, Corbin J. Basics of qualitative research. Sage publications; 1990..
33. Cohen J. A coefficient of agreement for nominal scales. Educational and psychological measurement. 1960 Apr;20(1):37-46.
34. Saunders J, Hume C, Timperio A, Salmon J. Cross-sectional and longitudinal associations between parenting style and adolescent girls’ physical activity. International journal of behavioral nutrition and physical activity. 2012 Dec;9(1):1-1.
35. Gibaud-Wallston J, Wandersman LP. Parenting sense of competence scale. Canadian Journal of Behavioural Science/Revue canadienne des sciences du comportement. 1978.
36. Gilmore L, Cuskelly M. Factor structure of the parenting sense of competence scale using a normative sample. Child: care, health and development. 2009 Jan;35(1):48-55.

37. Dix T. The affective organization of parenting: Adaptive and maladaptative processes. Psychological bulletin. 1991 Jul;110(1):3.

38. Morris AS, Silk JS, Steinberg L, Myers SS, Robinson LR. The role of the family context in the development of emotion regulation. Social development. 2007 May;16(2):361-88.

39. Zimmer-Gembeck MJ, Rudolph J, Kerin J, Bohadana-Brown G. Parent emotional regulation: A meta-analytic review of its association with parenting and child adjustment. International Journal of Behavioral Development. 2022 Jan;46(1):63-82.

40. Okorn A, Verhoeven M, Van Baar A. The importance of mothers’ and fathers’ positive parenting for toddlers’ and preschoolers’ social-emotional adjustment. Parenting. 2022 Apr 3;22(2):128-51.

41. Smith KE, Landry SH, Swank PR. The influence of early patterns of positive parenting on children's preschool outcomes. Early Education and Development. 2000 Mar 1;11(2):147-69.

42. Foster D, Rodrigues M, Somir I, Aziz T, Patel R, Ragunathan S, Sokolovic N, Jenkins J. Paternal positivity and child mental health: A meta-analysis. Journal of Child and Family Studies. 2022 Sep;31(9):2556-

Click here to access/download
Other
IRB Approval 23046 - Macalester College.pdf

Click here to access/download
Other
IRB Approval 23046 - Rollins College.pdf

Click here to access/download
Other
Consent Form - Pezalla and Davidson (2).pdf

Click here to access/download
Other
Debriefing Form - Pezalla and Davidson[65] (1).pdf

Click here to access/download
Other
Gentle_Parenting Questionnaire (1).pdf

Click here to access/download
Other
Recruitment Template - Pezalla and Davidson[31].pdf

Data File

Click here to access/download
Other
Gentle Parenting Dataset - PLOS ONE.sav

Revised Manuscript with Track Changes

1. "Trying to remain calm…but I do reach my limit sometimes": An exploration of the meaning of gentle parenting

~~2. AN EXPLORATION OF GENTLE PARENTING~~

~~3.~~2. Anne E. Pezalla[a] and Alice J. Davidson[b]

3. [a]Macalester College, St. Paul, MN, United States,

4. [b]Rollins College, Winter Park, FL, United States

5. Corresponding author: ~~ence concerning this article should be addressed to:~~

~~5.~~6. Email: ~~Anne E. Pezalla, Macalester College, St. Paul, MN, United States,~~ apezall1@macalester.edu

Formatted: Font: (Default) Arial, 11 pt

Formatted: Left

Formatted: Font: Not Italic

Formatted: Font: Not Italic

Formatted: Font: Not Italic

Formatted: Font: Not Italic

Formatted: Font: (Default) Arial, 11 pt

Formatted: Space Before: 0 pt, After: 0 pt

Formatted: Font: (Default) Arial, 11 pt

Abstract

Raising young children has always been hard, but evidence suggests that it may be getting harder. The isolation of the pandemic, the pressures to fulfill exacting parenting standards, and the explosion of "expert" parenting advice on social media have fueled the rise of "gentle parenting," an approach that pivots away from older, discipline-heavy parenting typologies and which promises the development of happier, healthier children. Despite the popularity of gentle parenting, it has received no empirical scrutiny. The current study represents the first systematic investigation of what gentle parenting entails. Data were gathered from a sample (N = 100) of parents of at least one child between the ages of 2 and 7 from the Midwest, Southeast, and Southwest. Approximately half (n = 49) of the sample identified as "gentle parents." Inductive analyses identified this approach as one that emphasizes high levels of parental affection and parents' and children's emotion regulation. Gentle parenting appears to be distinct from other established measures of parenting approaches in its emphasis on boundaries, yet the enactment of those boundaries is not uniform. Overall, gentle parents reported high levels of parenting satisfaction and efficacy, but a subset of gentle parents who were highly critical of themselves reported significantly lower levels of efficacy than the rest of the sample. Statements of parenting uncertainty and burnout were present in over one-third of the gentle parent sample. Implications are discussed for future research and increased support for those who identify as gentle parents.

Formatted: Left

Formatted: Font: 18 pt

Formatted: Font: (Default) Arial, 18 pt, Not Bold

~~"Trying to Remain Calm…But I do Reach my Limit Sometimes": An Exploration of the Meaning of Gentle Parenting~~

The COVID-19 pandemic left many parents around the world with a rare gift: less rushing around and more intentional time at home with their children (1). For many others, though, the pandemic was unilaterally bad. Evidence suggests that, for millions of parents, the pandemic increased parental stress (2) to levels that have not yet dropped to pre-pandemic levels (3) and lowered parents' feelings of efficacy in their parenting (4). Given the nearly impossible task of simultaneously juggling work responsibilities with caring for children at home due to the closure of schools and childcare facilities, it is no wonder that many parents of young children experienced poor mental health (5) and reported feelings of stress and exhaustion (6) during the pandemic. Those particularly vulnerable to the pandemic have been high-conflict families (1,7), families who employed harsh or inconsistent parenting (8), and families with children who were prone to stress or anxiety (9).

Evidence suggests that even beyond the pandemic, the work of rearing children is simply seen as more stressful and difficult than it was in the past. A 2023 study within the United States by the Pew Research Center (10) found that 41% of parents reported that being a parent is tiring, and 29% said it is stressful all or most of the time, with the youth mental health crises reported as a common concern among parents. Within the Pew report, 70% of mothers and 60% of fathers acknowledged that being a parent today is more demanding than it was just a few decades ago.

This spotlight on the suffering of parents is relatively new. Developmental Psychology, as a whole, has long focused on children (11), with parents "instrumentalized at the service of their children's development and best interest" (p. 8). Yet a focus on parents in their own right is merited, especially considering that *parental burnout*, which goes beyond the normality of parental stress and which severely and chronically overwhelms parents' resources to cope, is on the rise (12). Parental burnout appears to be more prevalent among parents who aim to be "perfect" (13), who are neurotic (14), and who have less social support (15). The question, then, is *what are these parents doing*?

To understand the current zeitgeist around parenting young children, it is helpful to consider the extent to which contemporary parenting approaches relate to arguably the most commonly-cited framework in parenting scholarship: Diana Baumrind's approaches. Baumrind's (16) theory-derived parent classification system, organized around the axes of parenting warmth and parenting discipline, originally generated three distinct approaches to

parenting: authoritative parenting was characterized by high parental warmth and discipline; authoritarian parenting was characterized by low parental warmth and high discipline; and permissive parenting was characterized by high parental warmth and low discipline. Maccoby and Martin (17) later expanded on Baumrind's three original parenting styles by adding a fourth type, neglectful parenting, which characterized parents who were low in both warmth and discipline. Broadly, investigations on these parenting styles highlight authoritative parenting as the optimal style for parents for its associations with wellbeing in children (18,19). Optimal parenting, according to this framework, is seen in those who employ lots of warmth and sufficient discipline and structure in their interactions with their children.

Baumrind’s typology~~ies~~, however, has~~have~~ not been immune from criticism, namely, that it is~~they~~ ~~are~~ Eurocentric, endorsing values that are limited to a primarily white, middle-class population (20), and that four relatively distinct parenting styles do not capture the nuance of parents’ lives, since most parents inevitably model a mixture of parenting styles (21). Gottman et al. (22) critiqued the parenting styles, noting that they provide little structure to parents in specific parent-child interactions. For example, what is a parent to do when a child throws a tantrum over a toy they cannot have? In such scenarios, and in many others, Baumrind’s authoritative style of high warmth and structure provides no clear guidelines. To address those limitations, Gottman and colleagues (22) proposed an alternative model that focuses on “emotion coaching,” which emphasizes parents’ explicit labeling of those emotions, awareness of and empathy for their child’s emotions, and problem solving with their child to deal with upsetting situations. Guided by the tenets of emotion coaching, a parent whose child is in the throes of a tantrum could address the child’s feelings (e.g., “you look very upset right now”), empathize with the child (e.g., “I’ve felt the same way as you before”), and then work on problem solving (e.g., “you can’t have that toy right now but maybe after we eat dinner”). Outcomes relating to parents’ use of emotion coaching have been largely positive, with emotion-coached children experiencing better self-regulation skills and less stress, although again, most of the focus has been on children (e.g., 23), and predominantly White European participants (24).

Still other parenting approaches include “positive parenting,” which emphasizes consistent and unconditional empathy and guidance (25), “conscious parenting,” (26), which focuses more on parents’ need for introspection and mindfulness in viewing their children as teachers of important life lessons, and most recently, “gentle parenting,” (27) which appears to be a catchall for variations of both “positive parenting” and “conscious parenting” and which centers on acknowledging a child’s feelings and motivations behind challenging behavior,

rather than correcting the behavior itself. Gentle parenting also prioritizes the notion of boundaries~~,~~ and giving children choices rather than orders. Gentle parenting appears to have arisen, at least in popular literature, as a rejection of the discipline- and hierarchical-laden nature of Baumrind's authoritative parenting typology. Speculations have also been shared that gentle parenting may be the embodied rejection of how parents were themselves raised when they were children. Indeed, the recent Pew Research Center (10) report found that 44% of respondents said they wanted to raise their children differently from their own upbringing, with many of those respondents reporting that they would be more gentle and less punitive.

Further complicating the array of parenting strategies and philosophies from which contemporary parents may~~have to~~ choose, is the fact that, unlike the above-mentioned parenting typologies, the idea of gentle parenting was not born of scholarship. Indeed, this approach seems to be fueled in great part by parenting "experts" and influencers on social media and YouTube. This reliance on social media platforms appears to have increased during the pandemic (28), and although evidence suggests that social media sources can provide helpful information about caregiving and can boost feelings of social connectedness to other parents (28,29), without discernment about the validity of parenting claims from so-called "experts," the consumption of this online content can be problematic, leading to heightened feelings of isolation and depression (30), especially among parents with higher anxiety (31). Child development researchers have not investigated the perspectives of gentle parents to determine a common definition of this approach, how this approach is related to other parenting typologies, the ways that gentle parents respond to specific challenges, and the sustainability of this approach from the perspective of the gentle parents themselves. Toward those ends, this exploratory study was guided by the following four research questions:

1. How do those who identify as "gentle parents" define such an approach?

Based on popular psychology descriptions of this new approach (27), we anticipated that "gentle parents" would have similar priorities as the "positive parenting" (25) and "conscious parenting" approaches (26), namely, emphasizing empathy and mindfulness.

2. How does "gentle parenting" relate to Baumrind's parenting typology? ?

Considering that "gentle parenting" seems to prioritize affection and to disavow punishment, we expected this approach to be more closely related to Baumrind's (16) authoritative and permissive parenting styles than to authoritarian or neglectful styles.~~.~~

3. How do gentle parenting ideologies relate to parenting behaviors when a child "misbehaves"?

4. To what extent is a gentle parenting approach associated with parent wellbeing?~~How are gentle parents doing?~~

Because this is the first empirical investigation of the “gentle parenting” approach, we had no guided hypotheses for the third and fourth research questions.

Method

Participants

Participants included 100 parents of at least one young child between the ages of 2 – 7 (M_{age} = 38.58 years, *SD* = 4.07; 84 females, 16 males). Participants resided in one of three metropolitan areas in the Midwest, Southeast, and Southwest in the US. They primarily identified as White (87%), with 7% identifying as Hispanic, 5% as Asian/Pacific Islander, and 1% as Black or African American. Participants were highly educated: 65% reported a postsecondary degree; 25% had a bachelor’s degree; and 10% had an associate’s degree, some college, or a high school diploma or equivalent.

Procedure

The study was approved by the authors’ institutional review boards (Macalester College served as the primary IRB of record [#23046]; Rollins College served as oversight IRB). These review boards were guided by the principles of The Belmont Report, federal regulations, state and local laws, and college policies. ~~.~~We distributed a link to a Qualtrics survey (www.qualtrics.com) via email to colleagues and personal acquaintances in various preschools, childcare facilities, and churches in the three metropolitan areas. We included a brief description of the study’s goal to explore parenting practices and challenges in 2023 in the wake of the COVID-19 pandemic. Our contacts shared information about the study and the survey link with individuals in their network who met our inclusion criteria of identifying as a parent of a currently 2- to 7-year-old child. Upon opening the survey link, participants provided written ~~their~~ informed consent after reading about the study’s purpose, the risks and benefits of participation, assurances of the confidentiality of their personal information and responses, the voluntary nature of the study, and their ability to withdraw at any time. The survey took approximately 20 minutes to complete (*M* = 22.6, *SD* = 15.4) and participants received a $10 Amazon gift card as compensation within 48 hours. Data collection commenced on July 25th, 2023, and ended on October 26th, 2023, at which time we exported participants’ responses

Formatted: Font: 18 pt

Formatted: Font: (Default) Arial, 18 pt, Not Bold

Formatted: Left

Formatted: Font: 16 pt

Formatted: Font: 16 pt

Formatted: Font: 16 pt

Formatted: Font: 16 pt

to SPSS (quantitative measures) and Excel (qualitative responses) for data cleaning, coding, and analysis. Of the 111 total individuals who completed the survey, we utilized all responses for establishing qualitative coding reliability. To ensure the credibility of responses, we ~~had~~ included three validation questions in the survey, which asked participants to select a particular response; 11 respondents ~~, but then deleted responses for 11 respondents who~~ failed thosree ~~validation~~ questions and thus we deleted their data for ~~before we conducted~~ our primary analyses.

Measures

Formatted: Font: 16 pt

Formatted: Font: 16 pt

Parenting Approaches~~*Adjectives*~~

We measured parenting approaches via a list of 16 parenting descriptors ~~and an open-ended question regarding participants' responses to their child's misbehavior~~ and open-ended responses to a question about how they responded to their child(ren)'s misbehavior and how they approach parenting. ~~.~~ Participants selected from a list of adjectives (accepting, affectionate, confrontational, conscious, consistent, expressive, firm, gentle, indulgent, intentional, permissive, reactive, respectful, responsive, strict, and warm) to describe how "your primary parental figure(s) approached parenting when you were a young child." They could select all that applied. Participants selected from the same list of adjectives to indicate their personal approach to parenting their 2- to 7-year-old child(ren). When they selected 'gentle,' they were prompted to describe "what does gentle mean to you as it applies to your personal approach to parenting?" Responses ranged from 3 to 77 words (M = 19.6, SD = 14.10). ~~To analyze these open-ended descriptions, we utilized an inductive approach (32), in which we first engaged in independent, close readings of the responses and open coding, making notes about any specific themes or descriptors used to illustrate what gentle parenting meant to participants. After discussing the emerging themes identified in the responses, we engaged in an iterative process of (a) developing and refining coding categories with operational definitions regarding parents' descriptions of their personal attempts to regulate their own emotions and to help their child regulate their emotions, (b) continued close readings of participants' gentle parenting descriptions, and (c) ongoing discussion about relevant examples and counterexamples in individual responses. We achieved strong inter-rater reliability for each coding category utilizing our final gentle parenting coding scheme (Cohen's κ > .80; see Table 1).~~

~~Parenting Themes in Narrative Prompts~~

Formatted: Font: 14 pt, Not Italic

Formatted: Font: 14 pt, Not Italic

Formatted: Font: 14 pt, Not Italic

Parents also responded to a prompt in which they were asked to: *Think about a recent time when your 2- to 7-year-old child was exhibiting challenging behavior (e.g., 'acting out,' misbehaving, or being disruptive at home or in public). How did you respond in that situation?* Responses ranged from 13 to 353 words (*M* = 73.91, *SD* = 70.39).

Additionally, parents described their approach to parenting. Responses to this prompt ranged from 1 to 348 words (M = 47.68, SD = 59.21). Similar to our method for coding the 'gentle' responses, we utilized an inductive approach consisting of close readings and open coding of the responses to identify emergent themes regarding self-identified gentle parents' responses to their children's misbehavior. Through multiple rounds of creating meaningful coding categories with operational definitions and discussion regarding what parents *do* when their children act out, we established a coding scheme consisting of 12 parenting responses (see Table 2). We engaged in several rounds of independent coding, discussion of disagreements, and refinement of the coding scheme until we achieved reliability of Cohen's κ > .80 for each category (33). We resolved all discrepancies and established the final codes for each response.

Parents also described their *approach to parenting*. Responses to this prompt ranged from 1 to 348 words (*M* = 47.68, *SD* = 59.21). We examined specific instances of parenting approach descriptions to capture a more holistic picture of the philosophy and practice informing gentle parents' responses to their child's misbehavior and to better understand gentle parents' wellbeing after conducting our primary analyses (See Results).

Parenting StylesQuantitative Measures

Formatted: Font: 14 pt, Not Italic

Formatted: Font: 14 pt, Not Italic

Parenting styles were measured using 197 items from the Parenting Style Scale (34), which was adapted from Baumrind's original typology. Items were scored on a 5-point Likert scale (1 = Never; 5 = Always) with 4 items measuring *indulgent (i.e., permissive) parenting* (e.g., "*I make decisions in consultation with my child.*"; α = .72), 4 items measuring *authoritative parenting* (e.g., "*I give my child reasons for my directions.*"' α = .73), 5 items measuring *authoritarian parenting* (e.g., "*I have the final say with my child.*"; α = .62) and 4 items measuring *neglectful parenting* (e.g., "*My child wins arguments with me.*"; α = .64). Items were averaged within each subscale, so individual scores could range from 1 to 5 for each parenting type. Given our interest in comparing gentle parenting to existing parenting styles, we excluded one item on the authoritative subscale that specifically described using a "gentle manner with my child." For the remaining 18 items, we conducted a principal components analysis to confirm the 4 parenting styles using an oblique rotation (direct oblimin) to simplify the factors and allow factors to be correlated. The resulting structure matrix indicated four clear factors that corresponded with the intended

parenting styles, with the exception of one item (“I tell my child how happy he/she makes me”) that did not load on any of the four factors (factor score < .4). We excluded that item from the indulgent subscale.

Parent Wellbeing

Parent wellbeing was operationalized using 15 items describing ~~To assess participants’~~ *self-efficacy in the parenting role* and *satisfaction with parenting* ~~, we utilized 15 items~~ from the Parenting Sense of Competence (PSOC) scale (35). Participants indicated their level of agreement with each item by indicating a number between 1 (strongly agree) and 6 (strongly disagree) that best corresponded to their sense of parenting self-efficacy (e.g., “*Being a parent is manageable, and any problems are easily solved*”) and satisfaction (e.g., “*I go to bed the same way I wake up in the morning, feeling I have not accomplished a whole lot*”). The 8 efficacy items were reverse scored so that high scores on both subscales indicated positive parental experience. To analyze the two-factor structure of the PSOC, we conducted a principal components analysis with an oblique rotation (direct oblimin) to simplify the factors and allow factors to be correlated. The resulting structure matrix clearly indicated a two factor structure for efficacy and satisfaction. One of the satisfaction items (“My mother/father was better prepared to be a good mother/father than I am,”) loaded strongly on the efficacy factor (factor score > .4), so we included the item as part of the efficacy subscale. An additional satisfaction item (“A difficult problem in being a parent is not knowing whether you’re doing a good job or a bad job”) did not load on either factor so we excluded it from the composite scores. Items were averaged for each subscale to create composite parenting self-efficacy (9 items) and parenting satisfaction (5 items) scores. Cronbach’s alphas were adequate (α = .77~~8~~ and .81~~77~~ for efficacy and satisfaction, respectively) and similar to those observed with a normative sample in Gilmore and Cuskelly (36).

Coding and Data Analytic Plan

To analyze participants’ open-ended descriptions of the meaning of ‘gentle’, we utilized an inductive approach (32), in which we first engaged in independent, close readings of the responses and open coding, making notes about any specific themes or descriptors used to illustrate what gentle parenting meant to participants. After discussing the emerging themes identified in the gentle responses, we engaged in an iterative process of (a) developing and refining coding categories with operational definitions regarding parents’ descriptions of their personal attempts to regulate their own emotions and to help their child regulate their emotions, (b) continued close readings of participants’ gentle parenting descriptions, and (c) ongoing discussion about relevant examples and

counterexamples in individual responses. We achieved strong inter-rater reliability for each coding category utilizing our final gentle parenting coding scheme (Cohen's $\kappa > .80$) (33).

We u~~U~~sed~~ing~~ a similar inductive approach for coding the ways in which parents responded to their child's misbehavior; this approach consisted~~ing~~ of an iterative process of close readings, open coding and creation of emergent themes. W~~, w~~e established a coding scheme consisting of 12 operationally defined parenting responses to their child's misbehavior, and~~. We~~ engaged in several rounds of independent coding, discussion of disagreements, and refinement of the coding scheme until we achieved strong reliability (Cohen's $\kappa > .80$) for each category. We resolved all discrepancies and established the final codes for each response (see Tables 1 and 2).

Table 1. Themes in the Meanings of Gentle Parenting, According to Gentle Parents (N = 49).

Coding Category (Inter-rater reliability; Frequency of responses; Percentage of responses)	Operational Definition	Example
Parent regulates own emotions		
Staying calm (κ = .96; N = 23; 47%)	Parent emphasizes their maintenance of a calm state, such as keeping voice at a low, soft tone; taking deep breaths; taking time to respond; no yelling, screaming, or threats	Having a moderate reaction - never getting too alarmed or being too permissive, always monitoring and adjusting expectations to the needs of the child and environment.
No physical punishment or punitive measures (κ = 1.00; N = 10; 20%)	Parent explicitly states that they refrain from hitting, spanking, or grabbing their child.	To me gentle refers to my response in frustrating or highly emotional situations with our daughter. We do not hit, spank, demean, or punish our child to make them feel like less of a person. Gentle refers to our discipline techniques and how we respect our child enough to make better decisions on her own. Even if we have to say something many times, we still are not aggressive towards our child to get the point across.
Parent helps child regulate their emotions		
Emotion validation (κ = 1.00; N = 15; 31%)	Parent attends to, respects, tries to understand, and validates child's feelings/needs. This includes listening with empathy.	Gentle means my children feel that they can come to me with an issue or concern and be received with patience and feel heard.
Emotion scaffolding (κ = 1.00; N = 7; 14%)	Parent uses child's emotional experiences as teachable moments for growth and self-understanding. This includes helping child learn coping skills, such as labeling emotions with words, and helping child "get to the root of her feelings." This also includes "processing" with children, and using clear communication with a rationale provided, or explaining the 'why' behind decisions.	Always providing a soft landing pad for him to explore his own thoughts and processes…gentle to me really means that he knows that I will consistently guide him without anger or frustration directed towards him as he learns to navigate the world and his place in it.
Parent shows affection (κ = 1.00; N = 13; 27%)	Parent shows loving emotional and physical affection to child (e.g., hugs); descriptions of being loving, kind, warm	Soft language, maybe a hug & sitting together for awhile

Table 2. Themes in Gentle Parents' (N = 49) Narratives of Response to Child's Misbehavior.

Coding Category (Inter-rater reliability; frequency; percentage)	Definition	Example
PARENT-DIRECTED RESPONSES		
Punish (κ = .96; N = 15; 31%)	Either taking away a privilege (e.g., screen time or treats) or administering unpleasant stimuli (e.g., yelling, grabbing their arm, forcing an apology) in response to misbehavior. Allowing a natural consequence that is unpleasant for the child.	I tell them to knock it off.<br><br>[I] separate and punish them,
Call them out (κ = 1.00; N = 15; 31%)	Telling child that their behavior is not appropriate. Directly addresses the misbehavior.	I tell them that that behavior is unacceptable.<br><br>I said firmly that her behavior wasn't okay. (Something like "This behavior needs to stop. It's hurtful and not helpful.")
Rationalize (κ = .96; N = 24; 49%)	Explaining the reasons why their behavior was not okay. This reasoning can occur during misbehavior and sometimes afterwards when processing/debriefing the incident.	Try to explain the circumstances ("although I read that toddlers can't rationalize so well").<br><br>I explain why changing his behavior was important. I tried to explain it in a way that reflected the importance in relation to him and those around him.
Redirect (κ = 1.00; N = 10; 20%)	Attempting to re-channel children's 'acting out' or misbehavior to something more productive/healthy; offering a positive alternative to misbehavior that does not involve bribery.	I tried to redirect to do something positive with her like have some cuddle time and read a book together.<br><br>I offer and model a replacement behavior.
Disengage (κ = .85; N = 3; 6%)	Explicitly stepping back from or leaving an emotionally intense scenario in an effort to stay calm. Usually manifested in walking away and/or taking a deep breath. Stating the need to "hold/honor a boundary" around their own wellbeing and prioritizing that boundary over their child's demands.	I had to walk away and disengage to send the message that the extreme behavior will not get a response.<br><br>I usually try to take a second to compose myself

CHILD-CENTERED OR CO-CONSTRUCTED RESPONSES

Show affection (κ = 1.00; N = 10; 20%)	Providing any physical affection (e.g., hugging) or emotional affection (e.g., "I tell her I love her").	Whenever there is a set-back in this process, we say “do you need a hug?<br><br>Reminded her I loved her.
Emphasize calm (κ = .96; N = 14; 26%)	Trying to encourage/assist their child in calming their bodies when they are upset. Working towards a calm goal-state in the child.	I usually try to help my child regain her calm (to regulate, suggest some deep breathing or naming the big emotion) and have her use more appropriate behaviors (calm tone or “tell me in words please so I may understand you” type of directives) to come up with a choice/resolution.
Let it fly (κ = 1.00; N = 19; 39%)	Letting the child “feel their feelings” without interruption or interference.	Waited until his fit was over.<br><br>I let them have their feelings about it.
Minimize hierarchy (κ = 1.00; N = 12; 25%)	Attempting to “get on child’s level”, either physically (e.g., bringing body at the same height of child, sitting with them) or emotionally (e.g., acknowledging feelings, validating feelings).	Sat my child down on a nearby chair, knelt down so we were on the same level, made physical contact & eye contact<br><br>I validated their experience
Bargain (κ = 1.00; N = 5; 10%)	Negotiating with or bribing the child (e.g., giving food or screens) under the assumption that their “misbehavior” stemmed from an unmet need. This can also be preemptive bribes to help elicit desired behavior.	Asked them what they needed in the moment, tried to provide that if it was appropriate to the situation.
Ask (κ = 1.00; N = 5; 10%)	Asking child to stop misbehaving (e.g., can you use your words?).	Typically we ask several times to stop.<br><br>Asked nicely for them to please stop said behavior (being mean to little brother, not listening when I ask him to do something, etc.)

In addition, we examined specific instances of parenting approach descriptions to capture a more holistic picture of the philosophy and practice informing gentle parents' responses to their child's misbehavior and to better understand gentle parents' wellbeing after conducting our primary analyses (See Results).

To address our four research questions, our data analysis involved a mix of descriptive statistics (frequencies of identified qualitative themes regarding the meaning of 'gentle', adjectives used to describe personal v. parents' approaches to parenting, and misbehavior responses) and independent samples *t*-tests to compare gentle v. not-gentle parents on parenting styles and wellbeing. To control for Type I error due to multiple comparisons, we applied a Bonferroni correction for nine pairwise comparisons: age, number of children, four parenting styles (authoritative, authoritarian, permissive, neglectful), parenting efficacy, parenting satisfaction, and total adjectives. Given these nine comparisons, and our experiment-wise alpha level of 0.05, the corrected alpha was set to .006 ($p <$ 0.05/0).

Results

How do t~~T~~hose w~~W~~ho i~~I~~dentify as "g~~G~~entle p~~P~~arents" d~~D~~efine s~~S~~uch an a~~A~~pproach?

To explore our first research question, we identified the primary themes that emerged in the definitions of gentle parenting reported by the 49 participants who selected "gentle" as an adjective to describe their personal approach to parenting. The most prominent theme involved parents' descriptions of regulating their own emotions (59.2%), which emphasized staying calm and/or avoiding physical punishment or any punitive measures with their child (see Table 1). For example, one gentle parent, a 42-year-old mother of two children, ages 7 months and 3 years, noted:

> To me, gentle refers to my response in frustrating or highly emotional situations with our daughter. We do not hit, spank, demean, or punish our child to make them feel like less of a person. Gentle refers to our discipline techniques and how we respect our child enough to make better decisions on her own. Even if we have to say something many times, we still are not aggressive towards our child to get the point across.

A secondary theme highlighted parents' descriptions of assisting their children in regulating their emotions (40.8%), which involved validating and listening to children's emotional experiences and/or scaffolding emotions, such that they used their child's emotional experiences as teachable moments for growth and self-understanding. This theme was evident in the response of another gentle parent, a 36-year-old mother of two children, ages 1 and 3, who described the approach as "helping my kids understand how their feelings can impact themselves and those around them so that they can build healthy coping skills." Finally, a third theme captured parents' descriptions of showing physical and/or emotional affection to their child (26.5%), such as one parent, a 38-year-old mother of three children, ages 3, 6, and 8, who commented that to her, gentle parenting involves "showing them affection that shows I care and love them."

Gentle parenting definitions were often multi-thematic. Thirty-four percent (n = 17) of the descriptions of gentle parenting in our sample referenced more than one emergent theme, such as this response from a 37-year-old father of three children, ages 2, 5, and 7 [with **bolded inserts** added to illuminate themes]: "Gentle parenting means mostly maintaining a lower energy level [**parent regulates own emotions]** and being respectful of my children's need for rest and relaxation [**parent regulates child's emotions]**", or this response, from a 39-year-old mother of two children, ages 3 and 5: "Gentle parenting means no harsh words [**parent regulates own emotions]** and soft touches [**shows affection].**" Responses that defined gentle parenting with only one theme (n = 32) were still richly descriptive (average word count of single-themed responses was 20 words), such as the following response:

> To me gentle refers to my response [**parent regulates own emotions]** in frustrating or highly emotional situations with our daughter. We do not hit, spank, demean, or punish our child to make them feel like less of a person. Gentle refers to our discipline techniques and how we respect our child enough to make better decisions on her own. Even if we have to say something many times, we still are not aggressive towards our child to get the point across. - *42-year-old mother of two children, ages 6 and 10.*

To further explore the meaning of 'gentle' to self-identified gentle parents, we analyzed additional descriptors participants indicated regarding their approach to parenting. When participants selected gentle to describe their own parenting, 94% also selected affectionate, 88% selected accepting, 86% selected warm, 82% intentional, 78% respectful, and 71% selected conscious. This exploration also involved a series of Pearson Chi-Square tests to compare gentle and non-gentle parents in terms of the frequency with which they selected specific adjectives (other than gentle) to describe their personal approach to parenting. Gentle parents were statistically more

likely to describe themselves as ‘expressive’ (rf = .57) than non-gentle parents were (rf = .35), $X^2(1, N = 100) = 4.80$, p = .028, but they did not differ from non-gentle parents in the frequency with which they selected any other adjectives to describe their approach to parenting. We also compared gentle and non-gentle parents on the total number of descriptors they used to describe their parenting approach. An independent samples t-test indicated that gentle parents selected significantly more adjectives overall to describe their own parenting (M = 8.65, SD = 2.06) in comparison to non-gentle parents (M = 6.94, SD = 1.83), $t(98) = 4.41$, $p < .001$. A paired samples t-test indicated that gentle parents also provided approximately 50% more adjectives to describe their own parenting approach in comparison to the number of adjectives they used to describe their own parents (M = 5.98, SD = 2.23), $t(48) = 9.70$, $p < .001$), describing themselves as being more accepting, affectionate, conscious, consistent, expressive, intentional, respectful, and warm, and less indulgent, reactive, and strict (see Figure 1). Importantly, these differences were not unique to gentle parents: non-gentle parents also described their own parenting style with more adjectives in comparison to the number of adjectives they used to describe how they were reared by their parents (M = 4.94, SD = 2.00), $t(50) = 7.32$, $p < .001$.

Figure 1. How do you describe your parenting approach vs. how did your parents raise you? Differences in adjectives among gentle parents (n = 49).

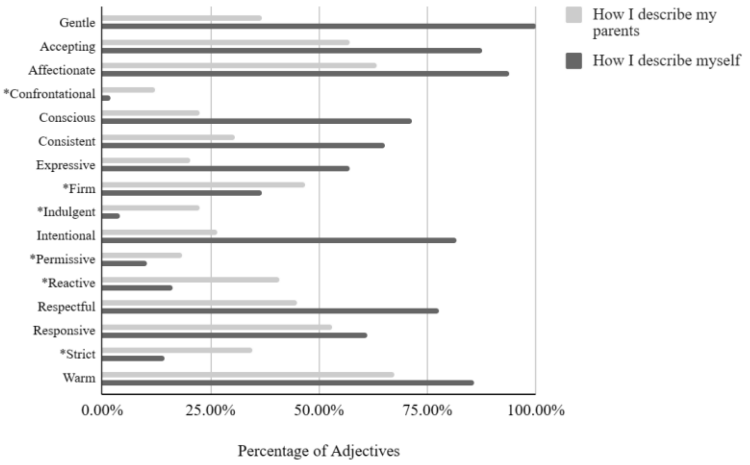

Note.* indicates a statistically significant ($p < .05$) difference in the percentage of adjectives that gentle parents (n = 49) used to describe their own parenting approach versus the number of adjectives used to describe how they were raised by their parents.

Formatted: Font: Italic

Formatted: Indent: Left: 0", Line spacing: single

Formatted: Font: (Default) Arial, 12 pt, Not Italic, Font color: Black, Not Highlight

How dDoes gGentle pParenting rRelate to Baumrind's parenting typology?oOther eEstablished pParenting tTypologies?

To examine our second research question, we conducted a series of independent samples *t*-tests to compare differences in authoritative, authoritarian, indulgent, and neglectful parenting between gentle and non-gentle parents. Compared to those who did not identify as gentle in their parenting approach, gentle parents had higher scores on authoritative and indulgent parenting, and lower scores on authoritarian and neglectful parenting, though these differences did not reach statistical significance with the Bonferroni corrections ($p < .006$) (see Table 3).

Formatted: Font: 16 pt
Formatted: Font: 16 pt
Formatted: Font: 16 pt
Formatted: Font: 16 pt
Formatted: Font: 16 pt
Formatted: Font: 16 pt
Formatted: Font: 16 pt
Formatted: Font: 16 pt
Formatted: Font: 16 pt
Formatted: Font: 16 pt
Formatted: Font: 16 pt
Formatted: Font: 16 pt
Formatted: Font: 16 pt
Formatted: Font: 16 pt
Formatted: Font: 16 pt
Formatted: Font: 16 pt
Formatted: Font: 16 pt
Formatted: Font: 16 pt
Formatted: Font: 16 pt
Formatted: Font: 16 pt
Formatted: Font: 16 pt
Formatted: Font: 16 pt
Formatted: Font: 16 pt
Formatted: Font: 16 pt
Formatted: Font: 16 pt
Formatted: Font: 16 pt
Formatted: Font: 16 pt

Commented [1]: @annie.pezalla@gmail.com , does this work?
Assigned to annie.pezalla@gmail.com

Table 3. Differences Between Gentle and Non-Gentle Parents in Baumrind's Parenting Approaches, Age, and Number of Children.

Variable	Gentle Parents (n = 49)		Not Gentle (n = 51)		t(98)	p	Cohen's d
Parenting Approach	*M*	*SD*	*M*	*SD*			
Indulgent	4.20	0.47	4.03	0.53	1.65	.051	.186
Authoritative	3.95	0.45	3.85	0.61	.745	.458	.152
Authoritarian	3.38	0.50	3.39	0.41	-.504	.615	.102
Neglectful	2.40	0.46	2.41	0.48	-.131	.896	.027
Age	39.02	3.83	38.16	4.27	1.063	.291	.213
Number of Children	2.10	0.77	2.12	0.65	.109	.913	.022

Formatted: Width: 11", Height: 8.5"

Formatted Table

~~When participants selected gentle to describe their own parenting, 94% also selected affectionate, 88% selected accepting, 86% selected warm, 82% intentional, 78% respectful, and 71% selected conscious. Gentle parents were statistically more likely to describe themselves as ‘expressive’ (*rf* = .57) than non-gentle parents were (*rf* = .35), X^2(1, N = 100) = 4.80, *p* = .028, but they did not differ from non-gentle parents in the frequency with which they selected any other adjectives to describe their approach to parenting. Gentle parents, however, selected significantly more adjectives overall to describe their own parenting (*M* = 8.65, *SD* = 2.06) in comparison to non-gentle parents (*M* = 6.94, SD = 1.83), *t*(98) = 4.41, *p* < .001. Gentle parents also provided approximately 50% more adjectives to describe their own parenting approach in comparison to the number of adjectives they used to describe their own parents (*M* = 5.98, *SD* = 2.23), *t*(48) = 9.70, *p* < .001), describing themselves as being more accepting, affectionate, conscious, consistent, expressive, intentional, respectful, and warm, and *less* indulgent, reactive, and strict (see Figure 1). Importantly, these differences were not unique to gentle parents: non-gentle parents also described their own parenting style with more adjectives in comparison to the number of adjectives they used to describe how they were reared by their own parents (*M* = 4.94, *SD* = 2.00), *t*(50) = 7.32, *p* < .001.~~

How d~~D~~o g~~G~~entle p~~P~~arenting i~~I~~deologies r~~R~~elate to p~~P~~arenting b~~B~~ehaviors w~~W~~hen a c~~C~~hild "m~~M~~isbehaves"?

To explore the ways in which gentle parents responded to instances of their child(ren)’s misbehavior, we first calculated the frequency with which parents employed each of the 12 parenting responses (see Table 2). Similar to the definitions of gentle parenting, the responses to misbehavior were multifaceted, often illustrating a number of approaches. On average, gentle parents employed three different responses in these moments of misbehavior from their children (*M* = 2.96, *SD* = 1.33), with rationalizing (47%) and letting it fly (36%) as the most common responses. The excerpt below illuminates this multifaceted response to misbehavior [**bolded inserts** added to illuminate the tagged code]:

> I have noticed that my responses differ in the situation (likely not good). At home - we take quite a bit of time to get low to the ground (at her level)], affirm her emotions ("I see you are upset right now") [**minimize hierarchy**], try to understand the cause (if it's not obvious), try to explain the circumstances (although I read that toddlers can't rationalize so well) [**rationalize**], and sometimes try to let her solve the

Formatted: Font: 16 pt

Formatted: Font: 16 pt
Formatted: Font: 16 pt
Formatted: Font: 16 pt
Formatted: Font: 16 pt
Formatted: Font: 16 pt
Formatted: Font: 16 pt
Formatted: Font: 16 pt
Formatted: Font: 16 pt
Formatted: Font: 16 pt
Formatted: Font: 16 pt
Formatted: Font: 16 pt
Formatted: Font: 16 pt
Formatted: Font: 16 pt
Formatted: Font: 16 pt
Formatted: Font: 16 pt
Formatted: Font: 16 pt
Formatted: Font: 16 pt
Formatted: Font: 16 pt
Formatted: Font: 16 pt
Formatted: Font: 16 pt
Formatted: Font: 16 pt
Formatted: Font: 16 pt
Formatted: Font: 16 pt
Formatted: Font: 16 pt
Formatted: Font: 16 pt
Formatted: Font: 16 pt
Formatted: Font: 16 pt
Formatted: Font: 16 pt
Formatted: Font: 16 pt
Formatted: Font: 16 pt
Formatted: Font: 16 pt

problem for herself (probably less often), offer acceptable choices for moving forward that result in an acceptable outcome for us ("do you want to do x this way or that way") **[redirect]**. Whenever there is a set-back in this process, we say "do you need a hug? Let's hug it out." **[show affection]** then proceed to the next step. - *42-year-old mother of one child, age 3.*

To further explore those responses to children's misbehavior, we categorized the 12 distinct parenting responses into what we perceived to be either parent-directed responses or child-directed responses. Parent-directed responses were those that were guided by the parent and that represented firmer *boundaries*, or demarcated rules and/or limits, between the parent and child. These responses included punishing, calling them out, rationalizing, redirecting, and disengaging. Child-directed responses were those that were more child-centered and prioritized giving the child more control, power, and/or attention; they also de-emphasized the importance of or minimized the boundaries between the parent and child, reflecting a more democratic parenting approach. These responses included showing affection, emphasizing calm, letting it fly, asking, minimizing hierarchy, and bargaining. Within each of the misbehavior narratives, participants received a binary score of either 0 or 1 for each of the 12 parenting responses, depending on whether that response was evident in the narrative. We then summed the total number of parent-directed responses and the total number of child-directed responses for each participant. Overall, gentle parents were equally likely to describe parent-directed (M = 1.37, SD = .88) and child-directed (M = 1.33, SD = 1.07) responses, $t(48)$ = .185, p = .854. There was, however, much variation among parents within these two categories.

To capture variation in the extent to which gentle parents favored a parent-directed or child-directed approach, we computed a difference score, subtracting the number of child-directed responses from the number of parent-directed responses, such that positive scores indicated participants' inclusion of more parent-directed responses in their misbehavior narratives, negative scores indicated more child-directed responses, and scores of 0 indicated that self-identified gentle parents described an equal mix of parent- and child-directed responses. Consistent with findings that gentle parenting was multi-thematic and multifaceted, there was variation in gentle parents' difference scores indicating that gentle parenting is practiced on a spectrum. Difference scores ranged from -3 (higher on child-directed misbehavior responses) to 3 (higher on parent-directed responses). Most gentle parents (67%) described a combination of parent-directed and child-directed responses. Twenty gentle parents had negative difference scores indicating misbehavior response descriptions that included more child-directed responses, 18

gentle parents had positive difference scores, indicating descriptions with more parent-directed responses, and 11 gentle parents had scores of 0 indicating an equal number of parent- and child-directed responses described in their misbehavior narratives.

The following narrative illustrates a misbehavior response from a parent who described using only child-directed strategies (difference score = -3) [**bolded inserts** added to illuminate the tagged child-directed code]:

> We try our best to stay calm when children are "acting out." We let them feel their feelings [**let it fly**], and if they're really upset, go with them to another room to calm their bodies down [**emphasize calm**]. Later in the day when they are calmed down, we talk through what happened and why they felt that way [**minimize hierarchy**], what they could do instead if they got physical. Sometimes we don't react the way we want as parents! We never yell, but are sometimes more firm or say things we didn't mean to say. In those situations, we apologize and talk. *-35-year-old mother of two children, ages 1 and 4.*

Throughout this mother's response, the emphasis is not on parent-governed rules or expectations, but rather, the focus is on the child's feelings, needs, and abilities to (later) process whatever misbehavior had occurred. Parent-directed boundaries are not emphasized in these child-directed responses.

Contrasting those child-directed strategies were those from parents who endorsed a parent-directed response to misbehavior, such as the following (difference score = + 3) [**bolded inserts** added to illuminate the tagged parent-directed code]:

> Last night, she was in the bathtub and kicking the water so that it was splashing out of the tub. I explained to her that she could kick smaller in the tub, but big kicks are for the pool [**call them out**]. Big kicks in the tub is getting water everywhere. I explained that if she kept splashing, bathtime would be over [**rationalize**]. She was laughing and continued to kick water out of the tub, so I started draining the water, picked her out of the tub, and gave her the towel [**punish**]. She got back in the tub, and I repeated, no, bathtime is over, picked her out of the tub, and put the towel around her. She got upset and was crying. I think I acknowledged that it's hard, but that she needs to do little kicks in the tub. *- 37-year-old mother of two children, ages 1 and 3.*

Throughout this mother's response, the emphasis is on boundaries–on clearly stated rules and/or limits, set and maintained by the parent–even when there was clear disagreement about those rules from the child.

Situated between those two distinct approaches to misbehavior were the gentle parents who equally referenced a combination of parent- and child-directed responses when they discussed how they responded to their child(ren)'s misbehavior. The following response generated a difference score of zero, since it included both punishing (a parent-directed response) and bargaining (a child-directed response) [**bolded inserts** added to illuminate the tagged parent- and child-directed code]:

> [I] Tell the child how to act, walking feet, inside voices. Since my child is young if we go somewhere I feel a behavior might happen I remind them of expected behavior at that location and if they do so what they will earn [**bargain]**. When they don't have expected behavior they don't get to earn privileges [**punish]**.
>
> - *33-year-old mother of one child, age 3.*

To what extent is a ~~How~~ ~~are~~ g~~G~~entle p~~P~~arenting approach associated with parent wellbeing?~~s~~ d~~Doing?~~

In the current study, we had no directional hypotheses about whether gentle parents would differ from non-gentle parents in terms of their parenting self-efficacy or satisfaction. To explore this potential variance, we conducted two-tailed, independent-samples *t*-tests to determine the extent to which mean differences in parenting efficacy and parenting satisfaction differed among those who identified as a gentle parent versus those who did not. Parenting self-efficacy did not differ significantly among gentle- versus non-gentle parents ($t(98)$ = 1.48~~45~~, p = .142~~151~~), although mean scores were higher among gentle- (M = 4.32~~28~~, SD = 0.61~~3~~) compared to non-gentle parents (M = 4.13~~4.0~~5~~9~~, SD = 0.65~~6~~). Gentle parents reported more satisfaction ($M = 4.15$, $SD = 0.96$) than did non-gentle parents ($M = 3.73$, $SD = 0.92$), although the difference did not reach statistical significance with the Bonferroni correction, $t(98) = 2.26$, $p = 0.026$.

In slight contrast to these quantitative findings was a theme that arose from the qualitative data. Within the narratives gentle parents provided, they often reflected on their wellbeing, openly questioning whether they were "doing it [parenting] right." To gain a better understanding of these self-evaluations, we studied the parenting approach descriptions parents provided and we identified additional, similar, evaluative reflections. In response to the question about parenting approaches, one gentle parent, a 38-year-old mother of three children, wrote simply: "I'm hanging on for dear life." Another, a 43-year-old mother of two children, ages 3 and 5, wrote that "I confess I feel I have no idea what I'm doing much of the time." Following this emergent theme, we read across all gentle

parents' narrative responses to identify any utterances of what we called *self-critique,* which we defined as moments of parenting uncertainty, insecurity, and/or doubts about personal parenting efforts/attempts. Overall, 34.7% of gentle parents' responses about their parenting approach or about how they handled their children's misbehavior contained unprompted elicitations of self-critique. Below are several examples of gentle parents' self-critique about their parenting approach or response to their child's misbehavior:

> I would describe it [my parenting approach] as still exploring. I have read ALL the parenting books, listened to the podcasts, followed the socials… and can't say I feel I've found any one "philosophy" that we adhere to all the time…I don't really feel like I know what I'm doing - so still exploring what "my approach" is. *-38-year-old mother of two children, ages 5 and 7.*

> As a stay at home mother I get easily overstimulated and overwhelmed all day every day. I very much look forward to nap time and bedtime because those are pretty much the only times I get to be alone. *-36-year-old mother of two children, ages 1 and 4.*

> In public…we tend to rush through the normal process, skip steps, with varying degrees of success. When/if they don't work, we are more likely to transition into bribing/negotiating - which we are aware are not recommended approaches. We have observed that we use food too often as the carrot, which we need to work on. *- 42-year-old mother of one child, age 3.*

Because of the prevalence of the self-critique theme in gentle parents' responses, we explored the extent to which parenting efficacy and parenting satisfaction differed among those gentle parents who self-critiqued versus those who did not. Among all gentle parents in our sample, parenting self-efficacy among those who engaged in self-critique (M = 3.96, SD = 0.46) was significantly lower ($t(47) = 2.69$, p = .01) than the parenting self-efficacy of those who did not engage in self-critique (M = 4.44, SD = 0.65). Parenting satisfaction among those who engaged in self-critique (M = 3.94, SD = 0.90) also was lower than the parenting satisfaction of those who did not engage in self-critique (M = 4.13, SD = 0.70), but this difference was not statistically significant, $t(47) = 0.79$, p = .434)

Discussion

Formatted: Font: 18 pt

Formatted: Font: 18 pt

Parenting young children has always been hard, but evidence suggests that it might be getting harder (10). The pressures to fulfill exacting parenting standards, and the explosion of "expert" parenting advice on social media have The forced isolation of the pandemic, coupled with the information overload on social media about the right or wrong ways to care for children, has left many parents questioning their moment-to-moment interactions with their family and leaving them with feelings of burnout (2). Such feelings may have emerged as many caregivers increased their time spent in active parenting (i.e., more play/learning activities) and decreased their time spent disciplining their young children during the lockdown period, a trend that has, notably, emerged across the income spectrum (1). Within this time of parenting angst, the idea of "gentle parenting" has gained popularity, especially on social media, as a promising new approach to raise happier, healthier children. Since the idea of gentle parenting was first introduced in 2015 by British author and parent Sarah Ockwell-Smith, coverage of this approach has skyrocketed online. At the time of this publication, a Google search on "gentle parenting" generates 1.687 million hits. That number will undoubtedly continue to climb.

Despite the popularity of this approach, there has been no empirical research about what gentle parenting actually means among those who endorse this approach; no systematic investigations to interrogate the extent to which gentle parenting is, in fact, a distinct approach from other, established typologies of parenting; no explorations to determine how gentle parents respond to challenging moments with their children; and equally important, no pointed inquiries to consider whether gentle parents are faring any better than the rest of us. This study represents a preliminary step to address those important questions.

The mMeaning of 'gGentle pParenting' aAccording to gGentle pParents

Forty-nine percent of our sample described their personal approach to parenting as 'gentle'. These gentle parents ranged from 32 to 51 years of age, and they were no more or less likely to have additional children than non-gentle parents. At least among the three US urban populations from which we sampled, gentle parenting appears to be a widely endorsed approach to parenting young children among millennials and Gen-Xers. Our analyses suggest that gentle parenting is nuanced and multi-thematic, especially in comparison to the ways these gentle parents were raised when they were children, but it is primarily an approach that prioritizes parents' and children's emotion regulation and a high amount of parental affection. Almost 60% of self-identified gentle parents defined 'gentle' as

Formatted: Font: 16 pt
Formatted: Font: 16 pt
Formatted: Font: 16 pt
Formatted: Font: 16 pt
Formatted: Font: 16 pt
Formatted: Font: 16 pt
Formatted: Font: 16 pt
Formatted: Font: 16 pt
Formatted: Font: 16 pt
Formatted: Font: 16 pt
Formatted: Font: 16 pt
Formatted: Font: 16 pt
Formatted: Font: 16 pt
Formatted: Font: 16 pt

regulating their own emotions, which involved maintaining a sense of calmness and a cool head, as well as avoiding any punitive actions, such as yelling or spanking, when it came to parenting their young children. Consistent with our expectations, t~~T~~his theme is ~~consistent with~~ similar to contemporary positive parenting movements that emphasize the importance of caregivers remaining ‘mindful’ (25) and ‘conscious’ (26). Parent emotion expression and regulation have long been considered integral to young children’s developing social and emotional competence (37). Parents provide the primary context in which children first learn about healthy (and unhealthy) emotion expression and management (38), and evidence suggests that parents with better emotion regulation skills exhibit more positive parenting behaviors and have children with better emotion regulation and fewer internalizing symptoms (39). Close to 41% of the self-identified gentle parents in our sample also defined ‘gentle’ as assisting their children with emotion regulation. These parents emphasized the importance of validating their child’s emotions through listening with empathy, understanding, and respect, as well as using their child’s emotional experiences as opportunities for nurturing intimacy and teaching coping skills and problem-solving. Both of these themes are consistent with Gottman and colleagues’ (22) model of emotion coaching, which affirms the role of caregivers’ empathetic engagement and awareness of emotions in themselves, as well their critical role in acknowledging and scaffolding their children’s emotional expressions. A third theme mentioned by around a quarter of gentle parents focused on showing physical and/or emotional affection to their child. These descriptions of hugging their child and being loving, kind, and warm are consistent with what ‘positive’ parenting endorses (e.g., 40), and such maternal and paternal behaviors are largely positive in terms of their correlates with child outcomes (41,42).

Associations between g~~G~~entle p~~P~~arenting and Baumrind’s typology~~oOther pParenting tTypologies~~

As to whether or not gentle parenting is truly a unique approach or is, figuratively, “old wine in new skins,” the evidence from this study suggests that, consistent with our expectations, gentle parenting is similar to Baumrind’s (1966) authoritative and indulgent parenting styles; based on the adjectives gentle parents used to describe their approach, it also seems to be similar to the positive parenting model (40,41). Still, this approach ~~but~~ likely merits its own qualitatively distinct category in that it emphasizes *boundaries* as a novel alternative to discipline. In our analyses of gentle parents’ responses to their children’s misbehavior, we noted that 28% included a reference to boundaries. “We try to hold consistent boundaries,” wrote a 39-year-old mother of two kids, ages 3 and

Formatted: Font: 16 pt

Formatted: Font: 16 pt

Formatted: Font: 16 pt

Formatted: Font: 16 pt

Formatted: Font: 16 pt

Commented [2]: I know I changed it to Baumrind, but what if we instead tweaked this sub-heading here in the discussion to read:

Associations between gentle parenting and existing parenting models

Formatted: Font: 16 pt

Formatted: Font: 16 pt

Formatted: Font: 16 pt

Formatted: Font: 16 pt

Formatted: Font: 16 pt

Formatted: Font: 16 pt

Formatted: Font: 16 pt

11. “We focus a lot on communicating boundaries,” wrote a 35-year-old father of one child, age 5. This emphasis on boundaries is distinct from other parenting approaches, but interestingly, gentle parents’ behaviors–or at least, their self-reports of their behaviors–showed a great deal of diversity in terms of how those boundaries were carried out in moments of their children’s misbehavior. About 40% of gentle parents in our sample demonstrated fairly clear, demarcated rules or boundaries with their children (what we referred to as a parent-directed approach); about 40% illustrated a more child-directed, democratic approach, with less-clear boundaries; and the remainder showed a combination of approaches, some that minimized boundaries and others that bolstered them. This diversity underscores the notion that gentle parenting is not a uniformly applied approach, but rather a multi-thematic philosophy and a multifaceted practice that is best conceptualized on a spectrum.

“I do the Opposite of my Parents”

A common sentiment expressed among the gentle parents in our sample was that they would do a better job at rearing their young children than their own parents had done with them. In comparison to their descriptions of their own parents, gentle parents described themselves as more accepting, affectionate, conscious, expressive, respectful, responsive, warm, and intentional. Indeed, “more, more, more” was the theme of these gentle parents. On average, they reported 50% more adjectives in describing their own parenting style than they used to describe how they were raised by their parents. These gentle parents would do more for their children. They would *be* more.

For some in our sample, the emphasis seemed to be about undoing what they perceived to be harmful parenting practices that they had experienced when they were children. For example, one gentle parent, a 43-year-old mother of three children, ages 8, 4, and 8 months, wrote that her approach to parenting was, quite simply, to “do the opposite of my parents. No spanking or physical punishment.” This desire to not only “do more” for their children but to “do differently” can be seen in the adjectives gentle parents selected to describe themselves versus their parents. Eighty-two percent of gentle parents in our sample described themselves as “intentional,” a stark contrast from the “reactive” descriptor that 42% of them ascribed to their parents. These findings are aligned with the recent report from the Pew Research Center (10), which revealed that 43% of the sampled parents wanted to raise their children differently than they had been raised, specifically with more love and compassion. This [perception of an] inter-generational divide in parenting values is meaningful, and represents an under-explored area of research in developmental psychology. Future research should examine the relationships between self-identified

gentle parents and their own parents to further elucidate the findings in our study that a subset of gentle parents were rejecting the reactive manner in which they were parented.

Gentle Parents are Not So Gentle on Themselves

As to how these gentle parents are doing, our data, again, portray a nuanced portrait. Overall, the gentle parents in this study reported high levels of parenting self-efficacy and satisfaction, suggesting that on the whole, they are doing quite well. But the emergent theme of self-critique, expressed by over one-third of gentle parents, and the findings that, among those self-critical gentle parents, the levels of self-efficacy were significantly lower, illuminates the need for more explorations and more support of these parents. One of the gentle parents in our sample, a 40-year-old mother of two children, wrote that her approach to parenting is about "Trying to remain calm…but I do reach my limit sometimes." Gentle parenting seems to represent an approach that is extraordinarily gentle for the children, but perhaps not-so-gentle for the parents themselves. Future research would benefit from examining gentle parenting in association with additional measures of parent wellbeing, including parenting stress and perfectionism.

Limitations and Future Research Directions

The limitations of this study include the relatively small, homogeneous sample. We wonder: Is gentle parenting a predominantly White, high socioeconomic status approach to parenting among mothers? Is gentle parenting a stressful experience (at least for some) because of its ideologies, or because of other, unexplored variables? Future research is merited with a more diverse sample, one that accounts for both parents' and child(ren)'s age and gender, the number of children in the family, and family structure, to explore the extent to which gentle parenting is defined and/or practiced similarly among other demographic groups. The current study also was limited in its means of data collection. Although our survey data were rich in detail, it was limited in that it only gathered parents' self-reports on their "general" responses, rather than "child-specific" responses; this approach may not reflect parents' authentic behaviors with their child(ren), and it may have masked important variability among parents who behave in specific ways with one child versus another. Observational data of parent-child interactions are needed, ideally in naturalistic settings. Finally, the current study was limited in its single-time-point assessment of gentle parenting. Especially considering that many gentle parents in this study were self-critical–

questioning the merits of this approach and even “overwhelmed all day every day”--longitudinal research is needed on the sustainability of this approach for the parents, as well as its potential impact on child development.

Conclusions and Practical Implications

Guidance about the “best” ways to rear small children has been around for a long time. What seems to be unique about the gentle parenting movement is that it has not been presented or advocated by scholars of human development; rather, it has largely been the product of social media. Considering that parents are increasingly stressed or burned out by their caregiving responsibilities (12), it is imperative that evidence-based guidance is made available to those who are interested in gentle parenting. What does this approach entail? How is it related to other parenting approaches? Is it a sustainable approach for caregivers? These are empirical questions, and they deserve empirical answers. The current study represents the first systematic investigation of a parenting approach widely endorsed by contemporary parents of young children.

References

Formatted: Left

Formatted: Font: 18 pt

Formatted: Font: (Default) Arial, 18 pt, Not Bold

1. Schmeer KK, Singletary B, Purtell KM, Justice LM. Family disruption and parenting during the COVID-19 pandemic. Journal of Family Issues. 2023 Jan;44(1):112-38.
2. Desimpelaere EN, Soenens B, Prinzie P, Waterschoot J, Vansteenkiste M, Morbée S, Schrooyen C, De Pauw SS. Parents' Stress, Parental Burnout, and Parenting Behavior during the COVID-19 Pandemic: Comparing Parents of Children with and without Complex Care Needs. Journal of Child and Family Studies. 2023 Dec;32(12):3681-96.
3. Adams EL, Smith D, Caccavale LJ, Bean MK. Parents are stressed! Patterns of parent stress across COVID-19. Frontiers in psychiatry. 2021 Apr 8;12:626456.
4. Xue A, Oros V, La Marca-Ghaemmaghami P, Scholkmann F, Righini-Grunder F, Natalucci G, Karen T, Bassler D, Restin T. New parents experienced lower parenting self-efficacy during the COVID-19 pandemic lockdown. Children. 2021 Jan 24;8(2):79.
5. Kerr ML, Rasmussen HF, Fanning KA, Braaten SM. Parenting during COVID-19: A study of parents' experiences across gender and income levels. Family Relations. 2021 Dec;70(5):1327-42.
6. Dawes J, May T, McKinlay A, Fancourt D, Burton A. Impact of the COVID-19 pandemic on the mental health and wellbeing of parents with young children: a qualitative interview study. BMC psychology. 2021 Dec;9(1):1-3.
7. Low A, Yu Y, Sim LW, Bureau JF, Tan NC, Chen H, Yang Y, Cheon B, Lee K, Bakermans-Kranenburg M, Tsotsi S. Maternal distress and parenting during COVID-19: differential effects related to pre-pandemic distress?. BMC psychiatry. 2023 May 29;23(1):374.
8. Ostrov JM, Murray-Close D, Perry KJ, Perhamus GR, Memba GV, Rice DR, Nowalis S. Parenting and Adjustment Problems among Preschoolers during COVID-19. Journal of child and family studies. 2023 Jan;32(1):93-109.
9. Tatsiopoulou P, Holeva V, Nikopoulou VA, Parlapani E, Diakogiannis I. Children's anxiety and parenting self-efficacy during the COVID-19-related home confinement. Child: Care, Health and Development. 2022 Nov;48(6):1103-11.
10. Minkin R, Horowitz JM. Parenting in America Today.

11. Mikolajczak M, Roskam I. Parental burnout: Moving the focus from children to parents. New directions for child and adolescent development. 2020 Nov;2020(174):7-13.
12. Mikolajczak M, Gross JJ, Roskam I. Parental burnout: What is it, and why does it matter?. Clinical Psychological Science. 2019 Nov;7(6):1319-29.
13. Sorkkila M, Aunola K. Risk factors for parental burnout among Finnish parents: The role of socially prescribed perfectionism. Journal of Child and Family Studies. 2020 Mar;29:648-59.
14. Lebert-Charron A, Dorard G, Boujut E, Wendland J. Maternal burnout syndrome: Contextual and psychological associated factors. Frontiers in psychology. 2018 Jun 5;9:885.
15. Séjourné N, Sanchez-Rodriguez R, Leboullenger A, Callahan S. Maternal burn-out: an exploratory study. Journal of reproductive and infant psychology. 2018 May 27;36(3):276-88.
16. Baumrind D. Effects of authoritative parental control on child behavior. Child development. 1966 Dec 1:887-907.
17. Maccoby EE, Martin JA. Socialization in the Context of Family: Parent-Child Interaction. W: PH Munsen (ed. tomu), EM Hetherington (ed. serii), Handbook of Child Psychology, t. 4, 1–101.
18. Baumrind D. The influence of parenting style on adolescent competence and substance use. The journal of early adolescence. 1991 Feb;11(1):56-95.
19. Pinquart M, Gerke DC. Associations of parenting styles with self-esteem in children and adolescents: A meta-analysis. Journal of Child and Family Studies. 2019 Aug 5;28:2017-35.
20. Sorkhabi N, Mandara J. Are the effects of Baumrind's parenting styles culturally specific or culturally equivalent?.
21. Kuppens S, Ceulemans E. Parenting styles: A closer look at a well-known concept. Journal of child and family studies. 2019 Jan 15;28:168-81.
22. Gottman JM, Katz LF, Hooven C. Parental meta-emotion philosophy and the emotional life of families: theoretical models and preliminary data. Journal of family psychology. 1996 Sep;10(3):243.
23. Dunsmore JC, Booker JA, Ollendick TH, Greene RW. Emotion socialization in the context of risk and psychopathology: Maternal emotion coaching predicts better treatment outcomes for emotionally labile children with oppositional defiant disorder. Social Development. 2016 Feb;25(1):8-26.

24. Dudeney J, Hawes D, Johnson A, Eisenberg N, Kohlhoff J. Emotion socialization and child conduct problems: A comprehensive review and meta-analysis.
25. Rodrigo MJ, Almeida A, Spiel C, Koops W. Introduction: Evidence-based parent education programmes to promote positive parenting. European Journal of Developmental Psychology. 2012 Jan 1;9(1):2-10.
26. Tsabary S. The conscious parent: Transforming ourselves, empowering our children. Hachette UK; 2014 Aug 28.
27. Ockwell-Smith S. The gentle parenting book: How to raise calmer, happier children from birth to seven. Piatkus; 2016 Mar 3.
28. Anderson M, Vogels EA. Americans turn to technology during COVID-19 outbreak, say an outage would be a problem.
29. Hooper A, Schweiker C, Kerch C. Social support in a parenting Facebook group during the COVID-19 pandemic. Family Relations. 2023 Apr;72(2):530-46.
30. Newsham G, Drouin M, McDaniel BT. Problematic phone use, depression, and technology interference among mothers. Psychology of popular media. 2020 Apr;9(2):117.
31. Drouin M, McDaniel BT, Pater J, Toscos T. How parents and their children used social media and technology at the beginning of the COVID-19 pandemic and associations with anxiety. Cyberpsychology, Behavior, and Social Networking. 2020 Nov 1;23(11):727-36.
32. Strauss A, Corbin J. Basics of qualitative research. Sage publications; 1990..
33. Cohen J. A coefficient of agreement for nominal scales. Educational and psychological measurement. 1960 Apr;20(1):37-46.
34. Saunders J, Hume C, Timperio A, Salmon J. Cross-sectional and longitudinal associations between parenting style and adolescent girls' physical activity. International journal of behavioral nutrition and physical activity. 2012 Dec;9(1):1-1.
35. Gibaud-Wallston J, Wandersman LP. Parenting sense of competence scale. Canadian Journal of Behavioural Science/Revue canadienne des sciences du comportement. 1978.
36. Gilmore L, Cuskelly M. Factor structure of the parenting sense of competence scale using a normative sample. Child: care, health and development. 2009 Jan;35(1):48-55.

37. Dix T. The affective organization of parenting: Adaptive and maladaptative processes. Psychological bulletin. 1991 Jul;110(1):3.

38. Morris AS, Silk JS, Steinberg L, Myers SS, Robinson LR. The role of the family context in the development of emotion regulation. Social development. 2007 May;16(2):361-88.

39. Zimmer-Gembeck MJ, Rudolph J, Kerin J, Bohadana-Brown G. Parent emotional regulation: A meta-analytic review of its association with parenting and child adjustment. International Journal of Behavioral Development. 2022 Jan;46(1):63-82.

40. Okorn A, Verhoeven M, Van Baar A. The importance of mothers' and fathers' positive parenting for toddlers' and preschoolers' social-emotional adjustment. Parenting. 2022 Apr 3;22(2):128-51.

41. Smith KE, Landry SH, Swank PR. The influence of early patterns of positive parenting on children's preschool outcomes. Early Education and Development. 2000 Mar 1;11(2):147-69.

42. Foster D, Rodrigues M, Somir I, Aziz T, Patel R, Ragunathan S, Sokolovic N, Jenkins J. Paternal positivity and child mental health: A meta-analysis. Journal of Child and Family Studies. 2022 Sep;31(9):2556-

Response to Reviewers

Manuscript: PONE-D-24-07088

Title: "Trying to Remain Calm…But I do Reach my Limit Sometimes": An Exploration of the Meaning of Gentle Parenting

Reviewer comment	Authors' response
1. Please ensure that your manuscript meets PLOS ONE's style requirements, including those for file naming. The PLOS ONE style templates can be found at https://journals.plos.org/plosone/s/file?id=wjVg/PLOSOne_formatting_sample_main_body.pdf and https://journals.plos.org/plosone/s/file?id=ba62/PLOSOne_formatting_sample_title_authors_affiliations.pdf	We have carefully gone through the PLOS ONE's style requirements and have fixed the following:<br>- title page formatting<br>- font size and location for Level 1, 2, and 3 headings<br>- Table and Figure formatting<br>- References
2. Thank you for stating the following financial disclosure: "Annie Pezalla received a $1250 faculty fund from Macalester College for this study. She used those funds to compensate participants."<br>Please state what role the funders took in the study. If the funders had no role, please state: ""The funders had no role in study design, data collection and analysis, decision to publish, or preparation of the manuscript.""	The funders had no role in the study design, data collection and analysis, decision to publish, or in preparation of the manuscript. We included this amended Role of Funder statement in our cover letter. Thank you for changing the online submission form on our behalf.
3. Please include your full ethics statement in the 'Methods' section of your manuscript file. In your statement, please include the full name of the IRB or ethics committee who approved or waived your study, as well as whether or not you obtained informed written or verbal consent. If consent was waived for your study, please include this information in your statement as well.	We have updated our ethics statement to specify that Macalester College served as the primary IRB of record; we also specified that the IRB approvals were guided by the principles of The Belmont Report, federal regulations, state and local laws, and college policies.<br><br>We also described that written consent was obtained for the survey data.
4. Please review your reference list to ensure that it is complete and correct. If you have cited papers that have been retracted, please include the rationale for doing so in the manuscript text, or remove these references and replace them with relevant current references. Any changes to the reference list should be mentioned in the rebuttal letter that accompanies your revised manuscript. If you need to cite a retracted article, indicate the article's retracted status in the References list and also include a citation and full reference for the retraction notice.	We have gone carefully through the reference list to ensure that it is in accordance with PLOS standards.

Lucky you---the deadly split--accept it is great and you need to clean this up. I have looked at the comments, they are good comments and I think fairly easy to correct. Can you please do that so we can see if that reviewer would be satisfied?<br><br>Isn't this always fun?	Thank you very much for your supportive comments on this manuscript. We have addressed the comments from Reviewer #1 and eagerly await your collective review.<br><br>In response to your question: Yes, this is fun! ·
Reviewer 1	
Overall, this study tackles an important topic. Indeed, in the wake of social media parenting, parents have been at times receiving mixed information about what is considered the best approach to parenting and managing difficult behavior in children. I believe this work is important but requires review before being published in this esteem journal. As detailed below, the authors should be more specific in explaining their analysis and adjust for errors, with some sections needing restructuring. The discussion merits to be reviewed to provide a more in-depth explanation of the interesting findings.<br><br>Introduction<br>1. The introduction is well-written but it would be helpful to have hypotheses or expectations rather than research questions solely, especially that the analyses involve both quantitative and qualitative analyses.	Thank you for your endorsement of our work. Thanks especially for your helpful feedback in making it better.<br><br>We have updated our introduction to include several expectations for the research questions based on existing popular psychology sources, but we kept our language purposefully broad there, using "expectations" and not the more traditional "hypotheses", since the study was truly exploratory and no empirical investigations had yet been conducted on gentle parenting before. We also clarified our fourth research question to reflect our specific interest in understanding the extent to which gentle parenting is related to parents' wellbeing.
Method<br>2. Were there any exclusion criteria? You mention in the procedure that "Of the 111 total individuals who completed the survey, we utilized all responses for establishing qualitative coding reliability, but then deleted responses for 11 respondents who failed three validation questions before we conducted our primary analyses.", what were those? These should be included as inclusion/exclusion criteria	We explained the validation questions, and then we updated our exclusion criteria to specify that anyone who failed those validation questions was excluded from primary analyses due to our concerns about the lack of credibility of their responses.
3. Also it is standard practice to describe the measures before summarizing the procedure. I suggest these sections are switched in order.	We appreciate this feedback. In the order we presented our methodology in the original manuscript, it was akin to explaining how to cook a recipe first, then explaining the ingredients necessary afterwards! That's weird, and it makes much more sense to switch the order of things!<br><br>However, in the context of this mixed methods paper, the cohesion and flow of information

	became confusing when we explored switching the order in the manner you suggested, since important context (e.g., participant recruitment) was explained *after* the measures. Therefore, we ended up keeping the original order of participants, followed by procedures, then measures.<br><br>We were concerned that retaining our original flow was indeed going against standard practice, but we did a quick search of other recently-published articles within PLOS and it looks like we're in pretty good company, with several (here, here, and here) listing their participants & procedures information before their measures.
4. You also mention that "Participants selected from the same list of adjectives to indicate their personal approach to parenting their 2- to 7-year-old child(ren)." Does that mean that parents could report on more than one child? What if their approach to parenting is not "general" but rather "child-specific," in other their answers might differ when asked to think about different children.	Great point. We added an explanation in our limitations section to note that our prompts to parents, which forced them to consider "general" rather than "child-specific" responses (if they had more than one 2- to 7-year-old child), may have masked important variability among parents.
5. I would create a separate "data analysis" section detailing the process of qualitative analysis rather than having it in the section describing the measures. This will make it clear to the reader how you will be using both types of analyses. As it stands it is not clear what the hypotheses are, thus unclear which inferential statistics you will be using	Thank you for this suggestion. We re-organized our Measures section to describe the parenting approaches, parenting styles, and parenting wellbeing measures specifically. In a separate Coding and Data Analytic Plan subsection that follows the Measures, we described our inductive approach to coding the qualitative responses, as well as our approach to conducting inferential statistics to examine several of our key questions. We believe this has resulted in a more cohesive presentation of our methodology.
6. Could you please further expand on how you adapted the Parenting Style Scale to only use 17 items? You cite Saunders et al. (2012) as your reference, I am assuming because they also adapted 22 items of Baumrind original scale; however they described the analysis run to ensure validity and reliability of the factor analysis. I do not see this in your manuscript.	Saunders et al. (2012) excluded 3 items from the original Baumrind measure based on their analysis of the underlying factor structure. We utilized the same 19 items as Saunders et al., but ended up excluding two items. Given our interest in comparing *gentle* parenting to existing parenting styles, we excluded one item on the authoritative subscale that specifically described using a "*gentle manner with my child.*" For the remaining 18 items, we conducted a principal components analysis to confirm the 4 parenting styles using an oblique rotation (direct oblimin) to simplify the factors and allow factors to be correlated. This analysis led us to drop an additional item ("*I*

	tell my child how happy he/she makes me") that did not load on any of the four factors (factor scores < .4). We have included a description of the principal components analysis that informed our decision-making in the measures section.
7. Same comment for the PSOC which usually comprises 17 items.	We additionally conducted a principal components analysis to confirm the 2 parental competence constructs using an oblique rotation (direct oblimin). The resulting structure matrix clearly indicated a two factor structure for efficacy and satisfaction. One of the satisfaction items ("*My mother/father was better prepared to be a good mother/father than I am,*") loaded strongly on the efficacy factor (factor score > .4), so we included that item as part of the efficacy subscale. An additional satisfaction item ("*A difficult problem in being a parent is not knowing whether you're doing a good job or a bad job*") did not load on either factor so we excluded it from the composite scores. Items then were averaged for each subscale to create composite parenting self-efficacy (9 items) and parenting satisfaction (5 items) scores. We have included this clarification in our measures section.
8. Overall this section merits restructuring to have separate sections for: participants, measures (qualitative and quantitative, procedure, data analysis/coding	Thank you for the feedback. Please see our responses to #3 and #5.
Results<br>9. When running so many t-tests you should correct for type I error.	Thank you for this feedback. To control for Type I error, we applied a Bonferroni correction for nine pairwise comparisons: age, number of children, four parenting styles (authoritative, authoritarian, permissive, neglectful), parenting efficacy, parenting satisfaction, and total adjectives. Given these seven comparisons, and our experiment-wise alpha level of 0.05, the corrected alpha was set to .006 ($p < 0.05/9$). We edited our language, and modified our conclusions, based on this Bonferroni correction.
10. "Gentle parents were statistically more likely to describe themselves as 'expressive' (rf = .57) than non-gentle parents were (rf = .35), X2(1, N = 100) = 4.80, p = .028, but they did not differ from non-gentle parents in the frequency with which they selected any other adjectives to describe their approach to parenting." – which tests were used?	Thank you for this question. We clarified the statistical tests we conducted to compare gentle v. non-gentle parents in their selection of adjectives to refer to their personal approach to parenting and their parents' approach. Specifically, we explicitly named the tests conducted for these descriptive analyses.

	"This exploration also involved a series of Pearson Chi-Square tests to compare gentle and non-gentle parents in terms of the frequency with which they selected specific adjectives (other than gentle) to describe their personal approach to parenting. Gentle parents were statistically more likely to describe themselves as 'expressive' (r_f = .57)…We also compared gentle and non-gentle parents on the total number of descriptors they used to describe their parenting approach. An independent samples *t*-test indicated that gentle parents selected significantly more adjectives overall to describe their own parenting…A paired samples *t*-test indicated that gentle parents also provided approximately 50% more adjectives to describe their own parenting approach in comparison to the number of adjectives they used…"
11. Same for the next set of results.	Please see our response to Results #10 above.
12. How Does Gentle Parenting Relate to Other Established Parenting Typologies? – you ran t-tests to compare Baumrind's parenting styles scores across the 2 groups gentle vs non gentle. However, was there a factor structure conducted for that scale after removing some of the items? If the 4-factor structure does not stand, then we cannot use these 4 parenting styles as a basis.	As noted in our response to Method #6, we utilized the same 19 items as Saunders et al. (2012), but ended up excluding two items: one item due to its mention of 'gentle' and one item that did not load onto one of the four factors after we ran a principal components analysis to confirm the 4 parenting styles.
13. "To further explore those responses to children's misbehavior, we categorized the 12 distinct parenting responses into what we perceived to be either parent-directed responses or child-directed responses" – how did you come up with 12? What was the coding system?	Thank you for these important questions. We specify our coding system in our revised manuscript, detailing that these 12 distinct parenting responses were generated from our initial, open-coding approach to parents' responses to their children's misbehavior.
14. "In response to the question about parenting approaches, one gentle parent, a 38-year-old mother of three children, wrote simply: "I'm hanging on for dear life." Another, a 43-year-old mother of two children, ages 3 and 5, wrote that "I confess I feel I have no idea what I'm doing much of the time." – I cannot but notice the age of both mothers, I think it is important to account for some many confounding variables, including age, in order to answer the question "How are Gentle Parents Doing?"	Excellent point. In our Discussion section, we added a caveat that age (both of parent and child(ren)) is an important variable to explore when considering what, exactly, explains the variance in gentle parents' feelings of stress.

Discussion<br>15. You start your discussion bringing in the COVID-19 pandemic and lockdown; however, you collected data in 2023. Some parents did not yet have their kids in 2019-2020 (lockdown years) so I am not sure how relevant this reference is. Many other factors have affected "parents questioning their moment-to-moment interactions with their family and leaving them with feelings of burnout," such as the worldwide economic crisis, social media pressure about parenting pressures, more so than COVID-19.	Thank you for sharing this important perspective. For parents of very young children, who had not yet been born during the onset of COVID-19 restrictions, other stressors were likely at play.<br><br>We've edited our discussion to omit those overt references to the COVID-19 pandemic and lockdown and instead focused more on social media pressures and "information overload".
16. Given that the authors differentiated between positive and gentle parenting in the introduction, it would be interesting to contrast the two in the discussion. In other word, what is the added value of the themes you highlighted in your results?	Thank you for this very good suggestion, specifically, for us to circle back to the various parenting models we referenced in the Introduction section and to reiterate those models in the Discussion section.<br><br>We've referenced the positive parenting model in our Discussion, noting that gentle parenting seems similar to the positive parenting model, as well as to Baumrind's authoritative and indulgent parenting styles, with the exception that gentle parenting differs from *all of them* in its emphasis on boundaries, which is a unique term that is not prioritized in other parenting models.
17. Comparing gentle parenting results to Baumrind's typology merits further expansion. The t-test analysis you conducted does not allow for the conclusion you made. It might actually be interesting to run a factor analysis on that questionnaire and then try to map the gentle parenting onto the factor structure you get.	Thank you for this comment. We believe we have clarified this with our elaboration of the principal components analysis we conducted with the Baumrind scale. With the exception of 2 items, the 4-factor structure held and we believe our t-tests comparing self-identified gentle v. not gentle parents on the 4 styles is appropriate. Further, our mixed methods approach to exploring the additional parenting descriptors endorsed by gentle (v. not gentle) parents and the comparison between descriptors applied to personal parenting approach v. parents' approach provides a more holistic picture of our emerging understanding of who gentle parents are. Based on our results, gentle parents were high on indulgent and authoritative parenting, moderate on authoritarian parenting, and low on neglectful parenting. In addition, these gentle parents selected *more* total adjectives to describe their parenting approach compared to non-gentle parents. Notably, compared to non-gentle parents, they were more likely to describe themselves as expressive parents. Given that

	this is a preliminary study, these differences certainly merit additional exploration. One conclusion is that gentle parents are embracing aspects of both authoritative and indulgent parenting (as expected), yet they are also distinguishing themselves from the more hierarchical-laden nature of the authoritative approach and rejecting the more rigid authoritarian approach that is common among their parents. We do not believe the gentle parenting approach defined by gentle parents (i.e., high parental emotion regulation, high support in child's emotion regulation, high warmth/affection) can be fully captured by the existing Baumrind typology.
18. Overall, the discussion should be reviewed to include possible confounding variables such as age, number of children in the family, family structure, couple level of support, employment history among others. The results are indeed very interesting but the discussion seems quite superficial.	Excellent point. We added these important considerations in our limitations and future research sections.
6. PLOS authors have the option to publish the peer review history of their article (what does this mean?). If published, this will include your full peer review and any attached files.	Yes!
Reviewer #2: The manuscript is incredibly well-written. It is clear that the authors were mindful of the research and its implications using both the quantitative data and the qualitative data in a back-and-forth display to reach conclusions and consensus. This was done extremely well.	Thank you for your supportive words about our paper!
