# Supplementary material for: “Trying to remain calm…but I do reach my limit sometimes”: An exploration of the meaning of gentle parenting
Source: PLoS One. 2024 Jul 31;19(7):e0307492. doi: 10.1371/journal.pone.0307492 (PMC11290678; doi:10.1371/journal.pone.0307492)
Supplement: Attachment — Submitted filename: Response to Reviewers FINAL.docx [file pone.0307492.s003.docx]

# Response to Reviewers

Manuscript: PONE-D-24-07088

Title: “Trying to Remain Calm…But I do Reach my Limit Sometimes”: An Exploration of the Meaning of Gentle Parenting

| **Reviewer comment** | **Authors’ response** |
| --- | --- |
| 1. Please ensure that your manuscript meets PLOS ONE's style requirements, including those for file naming. The PLOS ONE style templates can be found at  <https://journals.plos.org/plosone/s/file?id=wjVg/PLOSOne_formatting_sample_main_body.pdf> and  <https://journals.plos.org/plosone/s/file?id=ba62/PLOSOne_formatting_sample_title_authors_affiliations.pdf> | We have carefully gone through the PLOS ONE's style requirements and have fixed the following:   - title page formatting - font size and location for Level 1, 2, and 3 headings - Table and Figure formatting - References |
| 2. Thank you for stating the following financial disclosure: "Annie Pezalla received a $1250 faculty fund from Macalester College for this study. She used those funds to compensate participants."   Please state what role the funders took in the study.  If the funders had no role, please state: ""The funders had no role in study design, data collection and analysis, decision to publish, or preparation of the manuscript."" | The funders had no role in the study design, data collection and analysis, decision to publish, or in preparation of the manuscript. We included this amended Role of Funder statement in our cover letter. Thank you for changing the online submission form on our behalf. |
| 3. Please include your full ethics statement in the ‘Methods’ section of your manuscript file. In your statement, please include the full name of the IRB or ethics committee who approved or waived your study, as well as whether or not you obtained informed written or verbal consent. If consent was waived for your study, please include this information in your statement as well. | We have updated our ethics statement to specify that Macalester College served as the primary IRB of record; we also specified that the IRB approvals were guided by the principles of The Belmont Report, federal regulations, state and local laws, and college policies.  We also described that written consent was obtained for the survey data. |
| 4. Please review your reference list to ensure that it is complete and correct. If you have cited papers that have been retracted, please include the rationale for doing so in the manuscript text, or remove these references and replace them with relevant current references. Any changes to the reference list should be mentioned in the rebuttal letter that accompanies your revised manuscript. If you need to cite a retracted article, indicate the article’s retracted status in the References list and also include a citation and full reference for the retraction notice. | We have gone carefully through the reference list to ensure that it is in accordance with PLOS standards. |
| Lucky you---the deadly split--accept it is great and you need to clean this up. I have looked at the comments, they are good comments and I think fairly easy to correct. Can you please do that so we can see if that reviewer would be satisfied?  Isn’t this always fun? | Thank you very much for your supportive comments on this manuscript. We have addressed the comments from Reviewer #1 and eagerly await your collective review.  In response to your question: Yes, this is fun! 😊 |
| ***Reviewer 1*** |  |
| Overall, this study tackles an important topic. Indeed, in the wake of social media parenting, parents have been at times receiving mixed information about what is considered the best approach to parenting and managing difficult behavior in children. I believe this work is important but requires review before being published in this esteem journal. As detailed below, the authors should be more specific in explaining their analysis and adjust for errors, with some sections needing restructuring. The discussion merits to be reviewed to provide a more in-depth explanation of the interesting findings.  Introduction 1. The introduction is well-written but it would be helpful to have hypotheses or expectations rather than research questions solely, especially that the analyses involve both quantitative and qualitative analyses. | Thank you for your endorsement of our work. Thanks especially for your helpful feedback in making it better.  We have updated our introduction to include several expectations for the research questions based on existing popular psychology sources, but we kept our language purposefully broad there, using “expectations” and not the more traditional “hypotheses”, since the study was truly exploratory and no empirical investigations had yet been conducted on gentle parenting before. We also clarified our fourth research question to reflect our specific interest in understanding the extent to which gentle parenting is related to parents’ wellbeing. |
| Method 2. Were there any exclusion criteria? You mention in the procedure that “Of the 111 total individuals who completed the survey, we utilized all responses for establishing qualitative coding reliability, but then deleted responses for 11 respondents who failed three validation questions before we conducted our primary analyses.”, what were those? These should be included as inclusion/exclusion criteria | We explained the validation questions, and then we updated our exclusion criteria to specify that anyone who failed those validation questions was excluded from primary analyses due to our concerns about the lack of credibility of their responses. |
| 3. Also it is standard practice to describe the measures before summarizing the procedure. I suggest these sections are switched in order. | We appreciate this feedback. In the order we presented our methodology in the original manuscript, it was akin to explaining how to cook a recipe first, then explaining the ingredients necessary afterwards! That’s weird, and it makes much more sense to switch the order of things!  However, in the context of this mixed methods paper, the cohesion and flow of information became confusing when we explored switching the order in the manner you suggested, since important context (e.g., participant recruitment) was explained *after* the measures. Therefore, we ended up keeping the original order of participants, followed by procedures, then measures.  We were concerned that retaining our original flow was indeed going against standard practice, but we did a quick search of other recently-published articles within PLOS and it looks like we’re in pretty good company, with several ([here,](https://www.ncbi.nlm.nih.gov/pmc/articles/PMC9994707/) [here](https://journals.plos.org/plosone/article?id=10.1371/journal.pone.0280681), and [here](https://journals.plos.org/plosone/article?id=10.1371/journal.pone.0285984)) listing their participants & procedures information before their measures. |
| 4. You also mention that “Participants selected from the same list of adjectives to indicate their personal approach to parenting their 2- to 7-year-old child(ren).” Does that mean that parents could report on more than one child? What if their approach to parenting is not “general” but rather “child-specific,” in other their answers might differ when asked to think about different children. | Great point. We added an explanation in our limitations section to note that our prompts to parents, which forced them to consider “general” rather than “child-specific” responses (if they had more than one 2- to 7-year-old child), may have masked important variability among parents. |
| 5. I would create a separate “data analysis” section detailing the process of qualitative analysis rather than having it in the section describing the measures. This will make it clear to the reader how you will be using both types of analyses. As it stands it is not clear what the hypotheses are, thus unclear which inferential statistics you will be using | Thank you for this suggestion. We re-organized our Measures section to describe the parenting approaches, parenting styles, and parenting wellbeing measures specifically. In a separate Coding and Data Analytic Plan subsection that follows the Measures, we described our inductive approach to coding the qualitative responses, as well as our approach to conducting inferential statistics to examine several of our key questions. We believe this has resulted in a more cohesive presentation of our methodology. |
| 6. Could you please further expand on how you adapted the Parenting Style Scale to only use 17 items? You cite Saunders et al. (2012) as your reference, I am assuming because they also adapted 22 items of Baumrind original scale; however they described the analysis run to ensure validity and reliability of the factor analysis. I do not see this in your manuscript. | Saunders et al. (2012) excluded 3 items from the original Baumrind measure based on their analysis of the underlying factor structure. We utilized the same 19 items as Saunders et al., but ended up excluding two items. Given our interest in comparing *gentle* parenting to existing parenting styles, we excluded one item on the authoritative subscale that specifically described using a "*gentle manner with my child.*” For the remaining 18 items, we conducted a principal components analysis to confirm the 4 parenting styles using an oblique rotation (direct oblimin) to simplify the factors and allow factors to be correlated. This analysis led us to drop an additional item (“*I tell my child how happy he/she makes me*”) that did not load on any of the four factors (factor scores < .4). We have included a description of the principal components analysis that informed our decision-making in the measures section. |
| 7. Same comment for the PSOC which usually comprises 17 items. | We additionally conducted a principal components analysis to confirm the 2 parental competence constructs using an oblique rotation (direct oblimin). The resulting structure matrix clearly indicated a two factor structure for efficacy and satisfaction. One of the satisfaction items (“*My mother/father was better prepared to be a good mother/father than I am,*”) loaded strongly on the efficacy factor (factor score > .4), so we included that item as part of the efficacy subscale. An additional satisfaction item (“*A difficult problem in being a parent is not knowing whether you’re doing a good job or a bad job*”) did not load on either factor so we excluded it from the composite scores. Items then were averaged for each subscale to create composite parenting self-efficacy (9 items) and parenting satisfaction (5 items) scores. We have included this clarification in our measures section. |
| 8. Overall this section merits restructuring to have separate sections for: participants, measures (qualitative and quantitative, procedure, data analysis/coding | Thank you for the feedback. Please see our responses to #3 and #5. |
| Results  9. When running so many t-tests you should correct for type I error. | Thank you for this feedback. To control for Type I error, we applied a Bonferroni correction for nine pairwise comparisons: age, number of children, four parenting styles (authoritative, authoritarian, permissive, neglectful), parenting efficacy, parenting satisfaction, and total adjectives. Given these seven comparisons, and our experiment-wise alpha level of 0.05, the corrected alpha was set to .006 (*p <* 0.05/9). We edited our language, and modified our conclusions, based on this Bonferroni correction. |
| 10. “Gentle parents were statistically more likely to describe themselves as ‘expressive’ (rf = .57) than non-gentle parents were (rf = .35), X2(1, N = 100) = 4.80, p = .028, but they did not differ from non-gentle parents in the frequency with which they selected any other adjectives to describe their approach to parenting.” – which tests were used? | Thank you for this question. We clarified the statistical tests we conducted to compare gentle v. non-gentle parents in their selection of adjectives to refer to their personal approach to parenting and their parents’ approach. Specifically, we explicitly named the tests conducted for these descriptive analyses.  “This exploration also involved a series of Pearson Chi-Square tests to compare gentle and non-gentle parents in terms of the frequency with which they selected specific adjectives (other than gentle) to describe their personal approach to parenting. Gentle parents were statistically more likely to describe themselves as ‘expressive’ (*rf* = .57)...We also compared gentle and non-gentle parents on the total number of descriptors they used to describe their parenting approach. An independent samples *t*-test indicated that gentle parents selected significantly more adjectives overall to describe their own parenting…A paired samples *t*-test indicated that gentle parents also provided approximately 50% more adjectives to describe their own parenting approach in comparison to the number of adjectives they used…” |
| 11. Same for the next set of results. | Please see our response to Results #10 above. |
| 12. How Does Gentle Parenting Relate to Other Established Parenting Typologies? – you ran t-tests to compare Baumrind’s parenting styles scores across the 2 groups gentle vs non gentle. However, was there a factor structure conducted for that scale after removing some of the items? If the 4-factor structure does not stand, then we cannot use these 4 parenting styles as a basis. | As noted in our response to Method #6, we utilized the same 19 items as Saunders et al. (2012), but ended up excluding two items: one item due to its mention of ‘gentle’ and one item that did not load onto one of the four factors after we ran a principal components analysis to confirm the 4 parenting styles. |
| 13. “To further explore those responses to children’s misbehavior, we categorized the 12 distinct parenting responses into what we perceived to be either parent-directed responses or child-directed responses” – how did you come up with 12? What was the coding system? | Thank you for these important questions. We specify our coding system in our revised manuscript, detailing that these 12 distinct parenting responses were generated from our initial, open-coding approach to parents’ responses to their children’s misbehavior. |
| 14. “In response to the question about parenting approaches, one gentle parent, a 38-year-old mother of three children, wrote simply: “I’m hanging on for dear life.” Another, a 43-year-old mother of two children, ages 3 and 5, wrote that “I confess I feel I have no idea what I'm doing much of the time.” – I cannot but notice the age of both mothers, I think it is important to account for some many confounding variables, including age, in order to answer the question “How are Gentle Parents Doing?” | Excellent point. In our Discussion section, we added a caveat that age (both of parent and child(ren)) is an important variable to explore when considering what, exactly, explains the variance in gentle parents’ feelings of stress. |
| Discussion  15. You start your discussion bringing in the COVID-19 pandemic and lockdown; however, you collected data in 2023. Some parents did not yet have their kids in 2019-2020 (lockdown years) so I am not sure how relevant this reference is. Many other factors have affected “parents questioning their moment-to-moment interactions with their family and leaving them with feelings of burnout,” such as the worldwide economic crisis, social media pressure about parenting pressures, more so than COVID-19. | Thank you for sharing this important perspective. For parents of very young children, who had not yet been born during the onset of COVID-19 restrictions, other stressors were likely at play.  We’ve edited our discussion to omit those overt references to the COVID-19 pandemic and lockdown and instead focused more on social media pressures and “information overload”. |
| 16. Given that the authors differentiated between positive and gentle parenting in the introduction, it would be interesting to contrast the two in the discussion. In other word, what is the added value of the themes you highlighted in your results? | Thank you for this very good suggestion, specifically, for us to circle back to the various parenting models we referenced in the Introduction section and to reiterate those models in the Discussion section.  We’ve referenced the positive parenting model in our Discussion, noting that gentle parenting seems similar to the positive parenting model, as well as to Baumrind’s authoritative and indulgent parenting styles, with the exception that gentle parenting differs from *all of them* in its emphasis on boundaries, which is a unique term that is not prioritized in other parenting models. |
| 17. Comparing gentle parenting results to Baumrind’s typology merits further expansion. The t-test analysis you conducted does not allow for the conclusion you made. It might actually be interesting to run a factor analysis on that questionnaire and then try to map the gentle parenting onto the factor structure you get. | Thank you for this comment. We believe we have clarified this with our elaboration of the principal components analysis we conducted with the Baumrind scale. With the exception of 2 items, the 4-factor structure held and we believe our *t*-tests comparing self-identified gentle v. not gentle parents on the 4 styles is appropriate. Further, our mixed methods approach to exploring the additional parenting descriptors endorsed by gentle (v. not gentle) parents and the comparison between descriptors applied to personal parenting approach v. parents’ approach provides a more holistic picture of our emerging understanding of who gentle parents are. Based on our results, gentle parents were high on indulgent and authoritative parenting, moderate on authoritarian parenting, and low on neglectful parenting. In addition, these gentle parents selected *more* total adjectives to describe their parenting approach compared to non-gentle parents. Notably, compared to non-gentle parents, they were more likely to describe themselves as expressive parents. Given that this is a preliminary study, these differences certainly merit additional exploration. One conclusion is that gentle parents are embracing aspects of both authoritative and indulgent parenting (as expected), yet they are also distinguishing themselves from the more hierarchical-laden nature of the authoritative approach and rejecting the more rigid authoritarian approach that is common among their parents. We do not believe the gentle parenting approach defined by gentle parents (i.e., high parental emotion regulation, high support in child’s emotion regulation, high warmth/affection) can be fully captured by the existing Baumrind typology. |
| 18. Overall, the discussion should be reviewed to include possible confounding variables such as age, number of children in the family, family structure, couple level of support, employment history among others. The results are indeed very interesting but the discussion seems quite superficial. | Excellent point. We added these important considerations in our limitations and future research sections. |
| 6. PLOS authors have the option to publish the peer review history of their article (what does this mean?). If published, this will include your full peer review and any attached files. | Yes! |
| Reviewer #2: The manuscript is incredibly well-written. It is clear that the authors were mindful of the research and its implications using both the quantitative data and the qualitative data in a back-and-forth display to reach conclusions and consensus. This was done extremely well. | Thank you for your supportive words about our paper! |
