# Supplementary material for: “Trying to remain calm…but I do reach my limit sometimes”: An exploration of the meaning of gentle parenting
Source: PLoS One. 2024 Jul 31;19(7):e0307492. doi: 10.1371/journal.pone.0307492 (PMC11290678; doi:10.1371/journal.pone.0307492)
Supplement: Attachment — Submitted filename: Response to Reviewers 6.10.24.docx [file pone.0307492.s005.docx]

# Response to Reviewers

Manuscript: PONE-D-24-07088

Title: “Trying to Remain Calm…But I do Reach my Limit Sometimes”: An Exploration of the Meaning of Gentle Parenting

| **Reviewer comment** | **Authors’ response** |
| --- | --- |
| Thank you for this revision. However, I find it more difficult to follow. You need a map of what is coming as I was confused. You need to have a data analytic section that explains ALL of the analysis you will do. All of a sudden I have Chi ares and t tests.  Tables 1 and 2 are results -- not sure why they are in the method. I see you did reliability measures but I am not sure why they are there.  I have made comments on the second version where the changes show---if you have questions please email me. | Thank you for this very good feedback. Your perspective has helped us see a much better organization to this paper, and for that, we are immensely grateful.  Per your requirements, we’ve made substantial edits to our Methods section to more clearly explain how we measured the constructs associated with Research Questions 1, 2, 3, and 4.  We’ve then created a clearer Data Analysis subsection within our Methods where we explain how we analyzed the data we collected to address Research Questions 1, 2, 3, and 4. *This is our most substantial revision and we believe it provides readers with the “road map” of which you were asking.  Putting more of this information into our Method section, where it belongs, has meant that we needed to revise our Results section. Specifically, we did the following:   - We took out some of the explanations about analyses in our Results section, since that information is now properly situated in our Methods section. - We included reliability of our coding in our Results section, not in the Method section, since that information is more appropriate to reveal as a Result, not as a Method. - We made sure to get right to the point in presenting the findings for each research question, to make sure we’re really hammering home the findings associated with each research question. |
