# Supplementary material for: “Trying to remain calm…but I do reach my limit sometimes”: An exploration of the meaning of gentle parenting
Source: PLoS One. 2024 Jul 31;19(7):e0307492. doi: 10.1371/journal.pone.0307492 (PMC11290678; doi:10.1371/journal.pone.0307492)
Supplement: Attachment — Submitted filename: Response to Reviewers 6.29.24 (1).docx [file pone.0307492.s006.docx]

# Response to Reviewers

Manuscript: PONE-D-24-07088

Title: “Trying to Remain Calm…But I do Reach my Limit Sometimes”: An Exploration of the Meaning of Gentle Parenting

| **Reviewer comment** | **Authors’ response** |
| --- | --- |
| The most problematic for me is the lack of a comparison to "non-gentle" parents in the use of how they parent. Many of the comments were not gentle and I wonder if this is a social media driven issue. | Thank you for this important critique. We have now included a comparison between gentle- and non-gentle parents in terms of their responses to their children’s misbehavior.  We share your impression that “many of the comments were not gentle.” Yes! Isn’t that interesting? We are eager to share these findings because they illuminate how truly multifaceted and complex the “gentle parenting” ideology appears to be. Some who identify as “gentle” behave in gentle ways; others who identify as “gentle” do not.  This variability in gentle parents’ responses to their children’s misbehavior also suggests an important consideration: that the gentle parenting approach is aspirational and unsustainable 100% of the time, especially in emotionally-intense situations. |
| Pages 19 and 20 have the same quote to show emotional regulation and only one theme—can you find a different quote? Then you use it again in the table. Seems like you should be able to pull out some variety. Also, after a direct quote, it helps to have a concluding sentence to end the paragraph. | Excellent point. Thank you! We’ve included new quotes on both pages to show the variety of responses that illustrate emotion regulation among gentle parents; we’ve also added a concluding sentence to the end of the paragraph you mentioned. |
| Page 22--- selected affectionate, 88% selected accepting, 86% selected warm, 82% intentional, 78% respectful, and 71% selected conscious.; Only need selected the first time | Edited in manuscript. |
| Table 2. Differences Between Gentle and Non-Gentle Parents in Baumrind’s Parenting Approaches, Age, and Number of Children. Wouldn’t it be better to use a multiple regression, rather than independent t tests? Age and number of children do not add to the variance between the two groups ----however, there are so few differences you would end up reporting no difference which is what you get anyway | We have carefully considered your recommendation to use multiple regression instead of independent t-tests. However, we believe that independent t-tests are more appropriate here for the following reasons:   1. **Alignment with Research Questions:** Our research questions focus on comparing the means between two distinct groups (gentle and non-gentle parents). Independent t-tests are specifically designed for this purpose and provide direct answers to our questions. 2. **Interpretability and Complexity:** Our goal is to present our findings in a clear and interpretable manner. Independent t-tests offer simplicity in analysis and interpretation, which is crucial for effectively communicating our results to a broader audience in PLOS. 3. **Sample Size Considerations:** Given our sample size, independent t-tests provide sufficient power to detect meaningful differences between groups. Multiple regression would require a larger sample size to achieve the same level of power, which is not feasible in our case.   We appreciate your understanding and hope that our justifications adequately address your concerns. |
| However please take out  Compared to those who did not identify as gentle in their parenting approach, gentle parents had higher scores on authoritative and indulgent parenting, and lower scores on authoritarian and neglectful parenting, although differences did not reach statistical significance with the Bonferroni corrections (p < .0125) (see Table 2). This part simple reflects variation and I do not think it really reflects true differences—you could add it to future research is you think it might be important. | Deleted from manuscript |
| How do gentle parenting ideologies relate to parenting behaviors when a child "misbehaves"? You don’t name the third behavior. | Thank you for this critique. We can understand that our original phrasing could have been misinterpreted. In our last iteration to you, we were trying to convey that, on average, gentle parents’ responses to their child’s misbehavior were multifaceted: these parents didn’t just employ one approach (e.g., to punish) when their child misbehaved; they used a variety of approaches (e.g., redirecting, disengaging, bribing, etc.). On average, gentle parents’ responses illustrated about 3 distinct approaches. The most-commonly listed approaches were “rationalizing” and “letting it fly,” followed by “punishing” and “emphasizing calm” and “minimizing hierarchy,” but we didn’t list any of those 3rd-, 4th-, and 5th-ranked approaches in our narrative; we only listed the top two. So again, we can understand how it’s confusing to have stated that parents’ responses illustrated, on average, 3 approaches…but then we only shared the two most commonly-listed approaches.  Rather than to reiterate what we reveal in Table 3 (which shows the frequency of each of those parental responses), we’ve revised this passage to state that, on average, parents employed about 3 responses when their child misbehaved, “revealing a multifaceted approach to those moments.” |
| Then it seems that many of these types of behaviors are not “gentle parenting”------they lose it, the yell. Are you sure that you have defined a type? | Please see our point above about gentle parenting being multifaceted and, perhaps, aspirational, rather than an approach applied 100% of the time. |
| Among all gentle parents in our sample, parenting self-efficacy among those who engaged in self-critique (M = 3.96, SD = 0.46) was significantly lower (t(47) = 2.69, p = .01) than the parenting self-efficacy of those who did not engage in self-critique (M = 4.44, SD = 0.65). Parenting satisfaction among those who engaged in self-critique (M = 3.94, SD = 0.90) also was lower than the parenting satisfaction of those who did not engage in self-critique (M = 4.13, SD = 0.70), but this difference was not statistically significant, t(47) = 0.79, p = .434)  How are these different? | One finding reports differences in **parenting self-efficacy** among those who engaged in self-critique v. those who did not, and the other finding reports differences in **parenting satisfaction** between those engaged in self-critique v. those who did not.  As we operationalized in the Method section, self-efficacy in the parenting role refers to a parent’s belief in their ability to perform the parenting role successfully; satisfaction refers to enjoyment in the role. These two sub-scales are related but distinct factors that comprise a parenting sense of competence scale. |
